# Efficiency of Health Investment: Education or Intelligence?

**DOI:** 10.1002/hec.3356

**Published:** 2016-05-03

**Authors:** Govert e. Bijwaard, Hans Van Kippersluis

**Affiliations:** ^1^ Netherlands Interdisciplinary Demographic Institute (NIDI‐KNAW/University of Groningen)GroningenThe Netherlands; ^2^ IZABonnGermany; ^3^ Erasmus School of EconomicsErasmus University RotterdamRotterdamThe Netherlands; ^4^ Department of EconomicsChinese University of Hong KongHong KongChina; ^5^ Tinbergen InstituteAmsterdamThe Netherlands

**Keywords:** education, intelligence, health, multistate duration model

## Abstract

In this paper, we hypothesize that education is associated with a higher efficiency of health investment, yet that this efficiency advantage is solely driven by intelligence. We operationalize efficiency of health investment as the probability of dying conditional on a certain hospital diagnosis and estimate a multistate structural equation model with three states: (i) healthy, (ii) hospitalized, and (iii) death. We use data from a Dutch cohort born around 1940 that links intelligence tests at age 12 years to later‐life hospitalization and mortality records. The results indicate that intelligent individuals have a clear survival advantage for most hospital diagnoses, while the remaining disparities across education groups are small and not statistically significant. © 2016 The Authors. *Health Economics* Published by John Wiley & Sons Ltd.

## 1 Introduction

Health disparities across educational groups are widespread, and – strikingly – growing over time (Meara *et al.*, 2008). While there has been considerable progress in recent years in unraveling the direction of causality (Grossman, 2015), much less attention has been devoted to understanding the mechanisms through which the higher educated peers achieve their health advantage. As a result, there is no conclusive evidence about *why* the higher educated peers are healthier than their less‐educated peers (Cutler and Lleras‐Muney, 2008; Mazumder, 2012; Grossman, 2015).

One often cited hypothesis is that the higher educated peers are more efficient producers of health investment. This could be due to (i) ‘productive efficiency’or (ii) ‘allocative efficiency’. The former hypothesis posits that higher education leads to a higher marginal product of a given set of health inputs (Grossman, 1972; Michael and Becker, 1973). In simple terms, the higher educated peers understand the doctor better and use existing medical care more efficiently. The allocative efficiency hypothesis on the other hand argues that higher educated individuals choose different, more efficient inputs into health investment, typically thought to be caused by better health knowledge and a more receptive attitude towards new information (Rosenzweig and Schultz, 1981; Muurinen, 1982).

While there is empirical evidence that higher educated individuals are more efficient users of health investment in terms of both productive and allocative efficiency (see Grossman, 2006 for an excellent review), it is not established whether this is actually the result of education *per se*. This is surprising for two reasons. First, much of the reasoning why higher educated individuals would be more efficient users of health investment equally holds for intelligence. For example, understanding the doctor better and adhering to complex treatments may be driven by intelligence rather than education (Gottfredson and Deary, 2004).

Second, our reading of the literature on education and health outcomes is that at least half of the health disparities across educational groups are due to the selection of healthier and more able individuals into higher education (Conti and Heckman, 2010; Conti *et al.* 2010; Heckman *et al.* 2014; Bijwaard *et al.* 2015). 1The reasoning is also corroborated by studies exploiting compulsory schooling reforms to establish the causal effect of education on health outcomes, which unanimously show that the causal effect of education on health outcomes is either much smaller than the correlation suggests (Lleras‐Muney, 2005; Van Kippersluis *et al.* 2011; Meghir *et al.* 2013), or even entirely absent (Albouy and Lequien, 2009; Clark and Royer, 2013). Hence, in recent years, evidence is growing that the presumed health returns to education may be smaller than previously thought, which also raises the question to what extent it is the actual attainment of education that improves health investment efficiency.

In this paper, we aim to answer two questions. First: Is education associated with a higher efficiency of health investment? A higher efficiency of health investment implies a higher level of health given the health investment inputs chosen, either for the same inputs (productive efficiency) or using different inputs (allocative efficiency). We choose to focus on an objective health outcome – survival – and an objective health investment measure – a hospitalization for a given condition. We operationalize efficiency of health investment as *a lower probability of dying conditional on being admitted to the hospital for a given diagnosis*. While the data do not permit disentangling productive from allocative efficiency, the data do provide a unique opportunity to answer a second question: To what extent is intelligence driving the potential efficiency gains associated with education?

To the best of our knowledge, this second hypothesis is novel. Rejecting or non‐rejecting this hypothesis has important policy implications. If educational attainment increases the efficiency of health investment then learning itself and the associated knowledge have non‐monetary returns in terms of health and survival gains. If instead most of the efficiency gains derive from intelligence, this suggests that supply‐side interventions (e.g., longer consultation time, more explicit prescriptions for lower IQ individuals, or nudging) are more appropriate to reduce population disparities in health and survival.

The data used are from a Dutch cohort study of individuals born around 1940 that links intelligence tests at age 12 years to (i) follow‐up surveys including education, and (ii) administrative records regarding hospitalizations between 1995 and 2005 and mortality between 1995 and 2011. We use a theoretical model with a health‐state‐dependent utility function and stochastic mortality risk to formulate hypotheses. Testing the theoretical hypotheses requires estimating a multistate structural equation model with three states: (i) healthy, (ii) hospitalized, and (iii) death.

The results suggest that the higher educated individuals are more efficient users of health investment: They have a smaller probability to die within 1 year after hospital admittance even conditional on self‐reported health and previous diagnoses. However, when accounting for selection into education based on intelligence, most of the efficiency gain is removed. It is mostly intelligent people who have a survival advantage for a given hospital diagnosis. Only for people with respiratory diseases, we found large differences in survival by education even conditional on intelligence. However, at least part of these differences seems to be driven by milder respiratory conditions among the higher educated individuals. In sum, the survival advantage among higher educated individuals derives largely from intrinsic abilities like intelligence, and milder conditions, rather than higher educated individuals being more efficient users of health investment.

This paper is structured as follows. The related literature, theoretical framework, and hypotheses are introduced in Section 2. Section 3 presents the multistate structural equation model to test the theoretical hypotheses. In Section 4, the Brabant data and the linked register data on hospitalization and mortality are discussed. Section 5 presents the empirical results and robustness checks. Section 6 concludes and provides a discussion of the results.

## 2 Theoretical Framework

### 2.1 Related literature

There are two related strands of literature that our paper contributes to: (i) the association between education and the efficiency of health investment, and (ii) the relationship between ability, education, and health outcomes. We will discuss each of these literatures and our contributions to them briefly.

#### Education and efficiency of health investment.

There is a number of studies showing direct or indirect evidence that education is associated with a higher efficiency of health investment. Gilleskie and Harrison (1998) estimate a structural production model for self‐reported health and provide tentative evidence that suggests both productive and allocative efficiency are at work. Kenkel (1991; 1995) provides evidence in favor of productive efficiency. Indirect evidence for allocative efficiency is given by Goldman and Smith (2002), Goldman and Lakdawalla (2005), Lleras‐Muney and Lichtenberg (2005), and Glied and Lleras‐Muney (2008) who show that higher educated individuals adhere better to, and benefit more from, complex treatments for HIV and diabetes, and sooner adapt to evolving medical technologies, and Lange (2011) who shows that higher educated individuals process objective risk factors for cancer into their subjective probabilities. We contribute to this literature by testing whether the presumed efficiency gains of education are in fact driven by intelligence.

#### Intelligence, education, and health.

It has been recognized for a long time that intelligence influences educational attainment and that this gives rise to the famous ‘ability bias’ in the returns to education (Griliches, 1977). More recently, studies have also established a strong correlation between intelligence and health outcomes (Auld and Sidhu, 2005; Cutler and Lleras‐Muney, 2008; Kaestner and Callison, 2011). The fact that intelligence influences both education and health outcomes has led to a recent series of closely related papers that estimate the extent to which the association between education and health is driven by selection on basis of intelligence and non‐cognitive skills (Conti and Heckman, 2010; Conti *et al.* 2010; Bijwaard *et al.* 2015). Our contribution to this literature is twofold. First, we extend the structural equation model by Bijwaard *et al.* (2015) to a multistate duration model, where individuals can be healthy, hospitalized, or deceased. Second, we do not focus on health or mortality, but on the efficiency of health investment, which sheds lights on the mechanisms through which education affects health.

### 2.2 Theoretical background and hypotheses

To structure thoughts, we propose a stylized model, somewhat similar to Murphy and Topel (2006), in which individuals maximize a utility function of the form:

(1) $$\overset{\infty }{\underset{t=0}{\int }}U\left\{U_{H}\left[C(t),L(t)\right],U_{I}\left[C(t),L(t)\right]\right\}P^{\left(k\right)}(0,t)e^{-\mathrm{\rho t}}\mathrm{dt}\;k=0,1$$

where *U*[·] is the utility function with inputs from consumption *C*(*t*) and leisure *L*(*t*) and *P* ^(*k*)^(0,*t*) is the transition probability for educational level *k* from age 0 to *t*. We envision a model in which utility per period derived from consumption and leisure is health dependent: *U*_*H*_[·] is the utility when in good health, while *U*_*I*_[·] is the utility when hospitalized. 2Obviously, the individual will also face a budget and time constraint, but analyzing a fully fledged life cycle model is beyond the scope of this paper.


We assume that in adulthood, there are three different states: (i) being healthy (H), (ii) being hospitalized (I), and (iii) death (D), where utility in death is normalized to zero. Hence, the matrix of transition probabilities *P* is a three by three matrix where the first row contains the transition probabilities from healthy to healthy, hospitalized, and death {*P*_*HH*_,*P*_*HI*_,*P*_*HD*_}, the second row contains the transition probabilities from hospitalized to healthy, hospitalized, and death {*P*_*IH*_,*P*_*II*_,*P*_*ID*_}, and no transitions are possible after death.

We assume that the transition process between the states is a Markov process and that the transition intensities *λ*(·) are constant over an age interval of 1 year but depend on education. The transition rates from healthy to hospitalized (*λ*_*HI*_), hospitalized to health (*λ*_*IH*_), healthy to death (*λ*_*HD*_), and hospitalized to death (*λ*_*ID*_) jointly comprise a matrix of transition intensities

(2) $$M(t)=\left(\begin{array}{ccc} -(\lambda_{\mathrm{HI}}(t)+\lambda_{\mathrm{HD}}(t)) & \lambda_{\mathrm{HI}}(t) & \lambda_{\mathrm{HD}}(t)\\ \lambda_{\mathrm{IH}}(t) & -(\lambda_{\mathrm{IH}}(t)+\lambda_{\mathrm{ID}}(t)) & \lambda_{\mathrm{ID}}(t)\\ 0 & 0 & 0 \end{array}\right)$$

In turn, the transition probability matrix from age *s* to age *t* is given by the following:

(3) $$P(s,t)=\exp(M(s))=V\Lambda (t-s)V^{-1}$$

where *V* is the matrix of eigenvectors of *M*(*t*) and Λ is the exponentiated matrix of eigenvalues (see Section A in the online appendix and Kalbfleisch *et al*. (1983) for more details).

Hypothesis 1 (Education is associated with a higher efficiency of health investment.)

(4) $$\begin{array}{c} E\left[\left.P_{\mathrm{ID}}^{\left(1\right)}(t)-P_{\mathrm{ID}}^{\left(0\right)}(t)\right|X\right]<0\\ E\left[\left.P_{\mathrm{IH}}^{\left(1\right)}(t)-P_{\mathrm{IH}}^{\left(0\right)}(t)\right|X\right]>0 \end{array}$$

where $P_{\mathrm{ID}}^{\left(1\right)}(t)$ is the 1‐year transition probability from hospitalized to death for the higher educated individuals at age *t*, and $P_{\mathrm{ID}}^{\left(0\right)}(t)$ is the same transition probability for the lower educated. Likewise, $P_{\mathrm{IH}}^{\left(k\right)}(t)$ refers to the transition probability from hospitalized to healthy for the higher (*k* = 1) and lower educated (*k* = 0) individuals. 3The probability to remain in hospital, *P*_*II*_, is the complement of the two probabilities in (4) and is extremely low because the probability to be in hospital again after exactly 1 year is very low.w The matrix *X* includes extensive controls for pre‐existing health conditions, demographic characteristics, and hospital diagnoses.

In words, Hypothesis 1 in Eq. (4) entails that for a given hospital diagnosis, a given state of self‐reported health, and conditional on demographics and social background, the higher educated individuals have a lower probability of dying.

Hypothesis 2 (Conditioning on intelligence, education does not improve the efficiency of health investment) Individuals choose their educational attainment *E* in adolescence on the basis of their intelligence *θ* and other characteristics *X*_*E*_. Because both *θ* and *X*_*E*_ may additionally influence the transition probabilities, education is endogenous with respect to health investment. The central thesis of this paper is that the reason why higher educated individuals have a higher efficiency of investment derives at least partly from their higher intelligence. Therefore, we account for intelligence *θ* in the transition probabilities, and the empirical tests of Hypothesis 2 can be formulated as follows:

(5) $$\begin{array}{c} E\left[\left.P_{\mathrm{ID}}^{\left(1\right)}(t)-P_{\mathrm{ID}}^{\left(0\right)}(t)\right|X,\theta \right]=0\\ E\left[\left.P_{\mathrm{IH}}^{\left(1\right)}(t)-P_{\mathrm{IH}}^{\left(0\right)}(t)\right|X,\theta \right]=0 \end{array}$$

## 3 Methodology

Our empirical approach is an extension of the structural equation framework developed by Conti *et al.*(2010) and Bijwaard *et al.* (2015). The model consists of three parts: (i) a binary educational choice depending on latent intelligence and other covariates, (ii) potential outcomes depending on the choice of education, latent intelligence, and other covariates, and (iii) a measurement system for intelligence.

### Educational choice.

The binary indicator for education *E*_*i*_ is defined as 1 if individual *i* took any education beyond primary school, and 0 if not:

(6) $$E_{i}=\left\{\begin{array}{cc} 1 & \;\text{if}\;E_{i}^{*}\geq 0\\ 0 & \;\text{otherwise} \end{array}\right.$$

where we assume $E_{i}^{*}$ is an underlying latent utility, which is continuous and linear and depends on latent intelligence *θ*, and observed characteristics *X* ^*E*^:

(7) $$E_{i}^{*}=\gamma X_{i}^{E}+\alpha_{E}\theta_{i}+\upsilon_{\mathrm{iE}}$$

with *υ*_*E*_ being an error term independent of *X* ^*E*^ and *θ*. We assume that *υ*_*E*_ is normally distributed, which implies that we have a probit model for the educational choice. We fix the variance at 1 because the variance is not identified in a probit model.

### Multistate potential hazard outcomes.

The second part is the potential outcomes part, in which there are two potential outcomes depending on whether the individual chose to pursue education beyond primary school or not. Bijwaard *et al.* 2015) defined the potential outcomes in terms of the mortality hazard. Our extension is a multistate model with three states: (i) healthy, (ii) hospitalized, and (iii) death, leading to four transition rates (Eq. (2), and eight potential transition rates. We define $\lambda_{\mathrm{HD}}^{\left(1\right)}(t)$ as the mortality rate from the healthy state for an individual with education level beyond primary school (*E*_*i*_=1), and $\lambda_{\mathrm{HD}}^{\left(0\right)}(t)$ as the mortality rate from the healthy state for an individual with an education level equal to primary school (*E*_*i*_=0). Similar definitions are used for the other transition rates.

We assume a Gompertz proportional hazard model in age for the two potential mortality rates from healthy, which has been shown to be an accurate representation of mortality between the ages of 30 and 80 (e.g. Gavrilov and Gavrilova, 1991; Cramer, 2012). Both potential hazards depend on the latent ability *θ* and observed characteristics while healthy *X* ^*H*^:

(8) $$\begin{array}{cc} \lambda_{\mathrm{HD}}^{\left(0\right)}(t|X^{H},\theta ) & =\exp\left(a_{\mathrm{HD}0}t+\beta_{\mathrm{HD}0}X_{i}^{H}+\alpha_{\mathrm{HD}0}\theta_{i}\right)\\ \lambda_{\mathrm{HD}}^{\left(1\right)}(t|X^{H},\theta ) & =\exp\left(a_{\mathrm{HD}1}t+\beta_{\mathrm{HD}1}X_{i}^{H}+\alpha_{\mathrm{HD}1}\theta_{i}\right) \end{array}$$

with *t* age in years. The hazard of becoming hospitalized is assumed constant conditional on the individuals socio‐demographics, health, and previous health investments captured in *X* ^*H*^

(9) $$\begin{array}{cc} \lambda_{\mathrm{HI}}^{\left(0\right)}(t|X^{H},\theta ) & =\exp\left(\beta_{\mathrm{HI}00}+\beta_{\mathrm{HI}0}X_{i}^{H}+\alpha_{\mathrm{HI}0}\theta_{i}\right)\\ \lambda_{\mathrm{HI}}^{\left(1\right)}(t|X^{H},\theta ) & =\exp\left(\beta_{\mathrm{HD}10}+\beta_{\mathrm{HI}1}X_{i}^{H}+\alpha_{\mathrm{HI}1}\theta_{i}\right) \end{array}$$

Because the duration of stay in hospital is never longer than a few months, we define the transition rates from hospitalized in terms of days in hospital (*τ*). Both the mortality rate for the hospitalized, *λ*_*ID*_, and the recovery rates from the hospitalized‐state, *λ*_*IH*_, are assumed to be exponential. Thus, for *k*={*H*,*D*}, we have the transition rates

(10) $$\begin{array}{cc} \lambda_{\mathrm{Ik}}^{\left(0\right)}(\tau |X^{I},\theta ) & =\exp\left(\beta_{\mathrm{Ik}00}+\beta_{\mathrm{HI}0}X_{i}^{I}+\alpha_{\mathrm{Ik}0}\theta_{i}\right)\\ \lambda_{\mathrm{Ik}}^{\left(1\right)}(\tau |X^{I},\theta ) & =\exp\left(\beta_{\mathrm{Ik}10}+\beta_{\mathrm{HI}1}X_{i}^{I}+\alpha_{\mathrm{Ik}1}\theta_{i}\right) \end{array}$$

### 3.1 Measurement system for intelligence.

The final part of the model is the measurement equation, linking one or two intelligence scores *M*_*ik*_ (*k* = 1,2) linearly with the discrete points of support of latent intelligence *θ*:

(11) $$M_{\mathrm{ik}}=\delta_{k}X_{i}^{M}+\alpha_{M_{k}}\theta_{i}+\upsilon_{iM_{k}}$$

with $\upsilon_{M_{k}}$ independent of *X* ^*M*^ and *θ*. We assume that $\upsilon_{M_{k}}$ is normally distributed with variance $\sigma_{M_{k}}^{2}$. The full likelihood function is given in Appendix B.

Without additional restrictions on the distribution of the latent factors, the model is not identified. Identification of our model is closely related to the identification in a mixed proportional hazard model, where we assume, as is standard in the literature on duration models (Heckman and Singer, 1984), that the unobserved heterogeneity follows a discrete distribution with finite points of support. We assume a discrete distribution with three points of support for latent intelligence *θ*_*l*_,*l* = 1,2,3. This is similar to including unobserved heterogeneity in the transition rates that is correlated over the different rates, and for identification, the unobserved heterogeneity needs to have a finite mean. We restrict *θ* to have zero mean, that is, $\sum p_{l}\theta_{l}=0$, where *p*_*l*_ is the probability that *θ* = *θ*_*l*_. This restricts one of the three support points *θ*_3_ and from the restriction that the probabilities *p*_*l*_ sum up to one, the probability *p*_3_. One additional restriction of setting $\alpha_{M_{1}}=1$ ensures identification of all *α*'s.

After estimating the transition rates in (8), (9), and (10), we calculate the 1‐year transition probabilities using the one‐to‐one translation given by Eqs (2) and (3). Using the delta‐method and the derivative of the transition matrix, we can derive the variance–covariance of the components of the transition matrix. This allows testing the theoretical Hypotheses 1 (Eq. (4)) and 2 (Eq. (5)).

## 4 Data and Descriptive Statistics

The data are from a Dutch cohort born between 1937 and 1941. The survey was held in 1952 among 5823 pupils of the sixth (last) grade of primary schools in the Dutch province of Noord‐Brabant and hence is referred to as the ‘Brabant data’. Surveys on education in 1983 and 1993 had a response rate of around 45%, leaving 2998 individuals. Hartog (1989) investigated the non‐response for the 1983 survey and found no attrition bias in a wage analysis. The Brabant data are subsequently linked to hospitalization records for the years 1995–2005 inclusive, and the mortality register and municipality register for the years 1995–2011 inclusive. The hospital discharge register contains data on both inpatient and day care patients of all general and academic hospitals in the Netherlands. Because the administrative registers are available since 1995, only 86% of the 2998 individuals could be traced in the municipality register in 1995, leaving us with a final sample of 2579 individuals.

**Endogenous variables:** In the analysis, we distinguish between three states. Individuals are ‘healthy’if they are alive and non‐hospitalized, ‘hospitalized’if they are alive but hospitalized for at least 1 day, and ‘death’if they are not alive. In our sample, 409 individuals, or 16%, died during the period 1995–2011 (of which 14% died in hospital). Average number of hospital stays (with overnight stay) over the period 1995–2005 is 1, with more than 25% of the hospital admissions due to circulatory problems, 15% due to neoplasms, 11% due to digestive problems, and 5% due to respiratory problems.

Our main variable of interest is *education*, defined as the highest level of education attended, in two categories: (i) *primary education* (14%), including those who attended at most (extended) 4At the time, pupils had to stay in school for at least 8 years, or until they reached the age of 14 years. Because regular primary school only consisted of sixth grades, some schools offered an additional 2‐year extended primary school (‘vglo’). primary school and (ii) *above primary education* (86%), including those who attended lower vocational education, general secondary school, and higher education.

*Intelligence* is modeled as a latent variable. In the Brabant data, there are two separate measurements for intelligence, both measured in the final grade of primary school: (i) the IQ progressive matrices test, which focuses on mathematical ability and is a replication of the British test designed by Raven (1958), and (ii) a vocabulary test (picking synonyms). The timing of the intelligence test at age 12 years avoids possible reverse causality from education to intelligence (Deary and Johnson, 2010). The Raven test is considered to be a ‘pure’ measurement of problem solving abilities, as it does not require any linguistic or general knowledge (Dronkers, 2002).

**Control variables:** A fairly standard set of socio‐demographic control variables such *age*, sex (*male*), *birth rank*, and *family social class* is included in all equations. Family social class is measured in three categories from lowest to highest depending on father's occupation (see Bijwaard *et al*. 2015 for details). We additionally know whether the child had to work in the parent's farm or company, defining the binary indicator *Child works*.

Factors additionally influencing the measurements of intelligence, *X* ^*M*^, include *school type* and the *number of teachers*. Additional factors influencing the educational choice, *X* ^*E*^, include *repeat*, which defines the number of classes that children had to repeat, and finally the *teacher's advice* and the *preference of the parents* concerning the further education of the pupil (work without further education, vocational education, or general secondary education).

Finally, to test whether higher educated individuals are more efficient users of health investment, we intend to keep health status and the type of hospital diagnosis constant. Therefore, from the state healthy, the set of control variables *X* ^*H*^ includes *self‐reported health* in three categories (measured in 1993), whether *hospitalized* before during the observation period, and the *last diagnosis* in case of a hospitalization (neoplasm, circulatory, respiratory, or digestive system). From the state hospitalized, the set of control variables *X* ^*I*^ includes self‐reported health, whether it was a *repeated admittance*, whether it was an *acute* admission, and the main diagnosis of the admission. The categories of all variables are defined in Table 1, which also includes descriptive statistics.

**Table 1:** Descriptive statistics by education level

	Primary	Above primary	All
	14%	86%	
*Mortality*			
Died	23%	15%	16%
% of which died in hospital	16%	12%	14%
*Hospitalization*			
Number of hospital stays	1.1	0.9	0.9
Emergency (acute) entry	49%	43%	44%
Length of stay (days)	10.2	9.3	9.7
Repeated admittance	44%	38%	39%
*Intelligence*			
Raven p.m. test	96.29	103.05	102.04
Vocabulary test	94.16	102.73	101.42
*Diagnosis at admission*			
Neoplasm	11%	16%	15%
Circulatory	30%	25%	26%
Respiratory	11%	4%	5%
Digestive	12%	11%	11%
*Control variables*			
Male	61%	58%	58%
Birth Rank	2.82	2.44	2.50
Self‐reported health in 1993			
Poor	11%	9%	10%
Family socio‐economic status^a^			
Lowest	66%	47%	49%
Middle	23%	45%	41%
Highest	0%	3%	3%
Child works	37%	22%	24%
School religion^a^			
Roman Catholic	82%	74%	74%
Protestant	14%	19%	19%
Public	4%	7%	7%
Number of teachers	6.68	6.95	6.92
Repeat^a^			
No Repetition of grade	33%	66%	61%
Repeated once	37%	24%	26%
Repeated twice or more	24%	6%	8%
Teacher's advice^a^			
Continue primary school	49%	18%	23%
Lower vocational education	37%	35%	36%
Lower secondary education	3%	27%	23%
Higher secondary education	1%	15%	13%
Preference of the parents^a^			
Work in family company	16%	10%	11%
Paid work without vocational education	33%	7%	10%
Paid work with vocational education	11%	6%	7%
General secondary education	19%	65%	58%

a Because of missing values, percentages do not add up to 100%.

## 5 Results

### 5.1 Basic model without control variables

**Figure 1:**
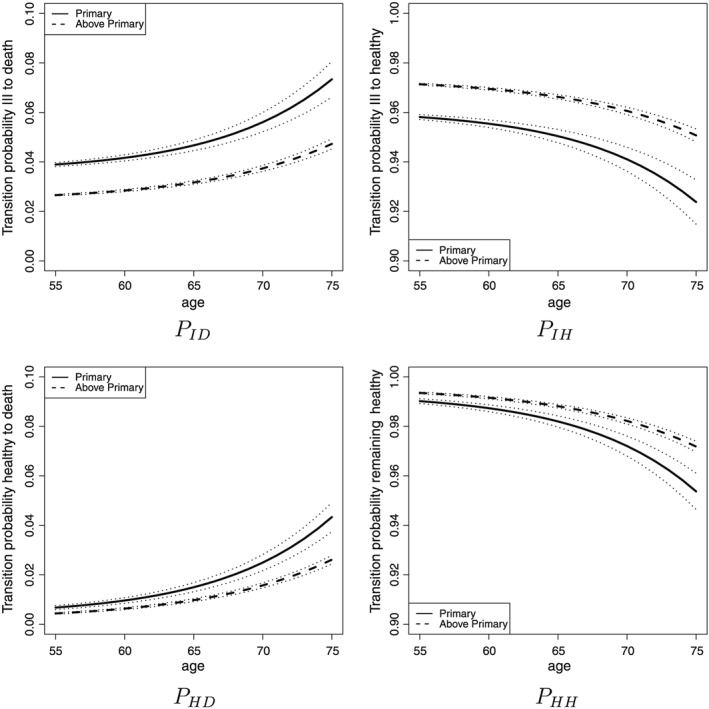
Transition probability over a 1‐year period (and the 95% confidence intervals) by age and education level (model without covariates)

To achieve a first impression of the impact of education on the efficiency of health investment, we start with estimating a basic model by education level without any control variables. The estimated parameters are reported in Table 2. Next, we calculate the implied transition intensities, and using (3), we calculate the transition probabilities for a 1‐year interval. In Figure 1, the four relevant transition probabilities are depicted. It is immediately clear that individuals who continued beyond primary education have a higher (lower) probability to recover (die) within 1 year of hospital admittance. Point estimates suggest that hospitalized individuals with a higher education have a 3% probability of dying, compared with 7% among the lower‐educated individuals. From the healthy state, the probability to die within 1 year is lower, and the probability to remain healthy is higher for higher educated individuals. All these differences are statistically significant. 5The formal tests of the significance of the difference in this basic model, the stratified model, and the structural model are reported in Table C.1 of the online appendix. This is an indication that our first hypothesis, education improves the efficiency of health investment, holds. In the remaining sections, we will only focus on the transition probability from hospital to death within 1 year, because the other transitions probabilities give very similar insights.

**Table 2:** Parameter estimates **simple** (no covariates included) stratified model by education level

	Primary education		Above primary	
*From healthy* a	To hospitalized	To death	To hospitalized	To death
(log) constant	−2.209	−12.609	−2.496	−12.122
	(0.050)	(1.794)	(0.023)	(0.846)
Age	—	0.126	—	0.112
		(0.027)		(0.013)
*From hospitalized* b	To healthy	To death	To healthy	To death
(log) constant	−2.357	−5.748	−2.255	−6.032
	(0.051)	(0.277)	(0.023)	(0.152)

a Duration time from healthy is years since birth.

b Duration time from hospitalized is days since hospital admission.

### 5.2 Stratified model including control variables

The previous analysis ignores that higher educated individuals have different characteristics than lower‐educated individuals and that the diagnosis at hospital admission may differ too. In this section, we include the control variables discussed in Section 4 but continue to assume that the education choice is exogenous (stratified models by education level). The results correspond to Eq. (4) in the theoretical framework. Table 3 reports the coefficient estimates.

**Table 3:** Parameter estimates **stratified** model by education level

	Primary education		Above primary	
***From hospitalized*** ^a^	To healthy	To death	To healthy	To death
Male	−0.100	−0.124	0.092	0.128
	(0.114)	(0.589)	(0.050)	(0.335)
Child is working – base is ‘No’				
Yes	−0.428^∗∗^	0.299	0.077	−0.736
	(0.117)	(0.650)	(0.056)	(0.453)
Missing	0.072	−0.017	−0.069	−1.081
	(0.220)	(1.162)	(0.085)	(0.667)
Birthrank ‐ base is ‘First’				
Second	0.059	—	−0.071	−0.918^+^
	(0.194)		(0.072)	(0.451)
Third or fourth	0.004	—	−0.169^∗∗^	−0.009
	(0.177)		(0.067)	(0.491)
Fifth or higher	0.007	—	−0.201^∗∗^	0.261
	(0.183)		(0.065)	(0.461)
Missing	−0.190	—	−0.380^∗∗^	0.664
	(0.273)		(0.122)	(0.844)
Health status in 1993 – base is ‘good’				
Poor health	0.077	0.274	−0.172^∗∗^	−0.204
	(0.129)	(0.630)	(0.064)	(0.435)
Previous hospitalization and last diagnosis				
Repeated admittance	−0.036	1.127	−0.110^+^	0.556
	(0.107)	(0.783)	(0.047)	(0.338)
Neoplasm	−0.331	1.419^+^	−0.313^∗∗^	2.695^∗∗^
	(0.187)	(0.657)	(0.069)	(0.502)
Circulatory	0.044	0.645	−0.033	0.692
	(0.139)	(0.796)	(0.061)	(0.580)
Respiratory	−0.413^+^	—	0.145	1.545^∗∗^
	(0.200)		(0.118)	(0.739)
Digestive	0.069	—	0.263^∗∗^	−1.317
	(0.168)		(0.079)	(0.675)
Acute	−0.428^∗∗^	1.270	−0.365^∗∗^	1.410^∗∗^
	(0.106)	(0.778)	(0.049)	(0.361)
(log) constant	−1.772	−8.183	−1.912	−8.736
	(0.211)	(1.158)	(0.072)	(0.709)
***From healthy*** ^a^	To hospitalized	To death	To hospitalized	To death
Male	−0.212	0.727^∗∗^	0.179^∗∗^	0.717^∗∗^
	(0.109)	(0.289)	(0.049)	(0.137)
Child is working ‐ base is ‘No’				
Yes	0.071	0.124	0.136^+^	0.240
	(0.114)	(0.272)	(0.055)	(0.147)
Missing	−0.352	−1.124	−0.114	0.181
	(0.213)	(0.617)	(0.083)	(0.200)
Family socio‐economic status ‐ base is ‘Low’				
Middle	−0.084	−0.097	−0.040	0.135
	(0.133)	(0.328)	(0.049)	(0.128)
High	−0.084	−0.097	0.217	0.540
	(0.133)	(0.328)	(0.131)	(0.318)
Missing	−0.492^+^	−0.787	0.013	0.377
	(0.244)	(0.674)	(0.123)	(0.318)
Birthrank ‐ base is ‘First’				
Second	0.175	−0.589	0.016	−0.130
	(0.185)	(0.409)	(0.071)	(0.176)
Third or Fourth	0.453^∗∗^	−0.328	0.026	−0.195
	(0.165)	(0.375)	(0.066)	(0.167)
Fifth or higher	0.385^+^	−0.074	0.073	−0.312
	(0.170)	(0.357)	(0.065)	(0.171)
Missing	0.768^∗∗^	0.848	0.027	−0.681
	(0.292)	(0.655)	(0.131)	(0.374)
Health status in 1993 ‐ base is ‘good’				
Poor health	0.419^∗∗^	−0.604	0.445^∗∗^	0.317
	(0.149)	(0.519)	(0.066)	(0.189)
Missing	−0.121	0.420	0.042	0.186
	(0.123)	(0.294)	(0.053)	(0.137)
Hospitalization and last diagnosis				
Has been in hospital	1.194^∗∗^	0.663^+^	1.351^∗∗^	0.988^∗∗^
	(0.135)	(0.299)	(0.056)	(0.142)
Neoplasm	0.789^∗∗^	1.576^∗∗^	1.109^∗∗^	1.659^∗∗^
	(0.218)	(0.537)	(0.082)	(0.176)
Circulatory	0.404^∗∗^	0.114	0.444^∗∗^	0.302
	(0.161)	(0.428)	(0.071)	(0.187)
Respiratory	1.047^∗∗^	1.696^∗∗^	0.475^∗∗^	0.143
	(0.208)	(0.491)	(0.141)	(0.421)
Digestive	0.148	−0.060	−0.155	−0.186
	(0.207)	(0.557)	(0.110)	(0.278)
(log) constant	−2.992	−13.325	−3.318	−11.584
	(0.189)	(1.933)	(0.069)	(0.902)
Age	−	0.125	−	0.087
		(0.028)		(0.014)

^a^Duration time from healthy is years since birth.^+^ *p* < 0.05 and^∗∗^ *p* < 0.01.

Table 3 indicates that individuals admitted with neoplasms are more likely to die (especially the higher educated individuals) and less likely to recover. Respiratory diseases also lead to less recovery and higher mortality. An emergency‐admittance to the hospital increases the mortality and decreases the recovery.

**Figure 2:**
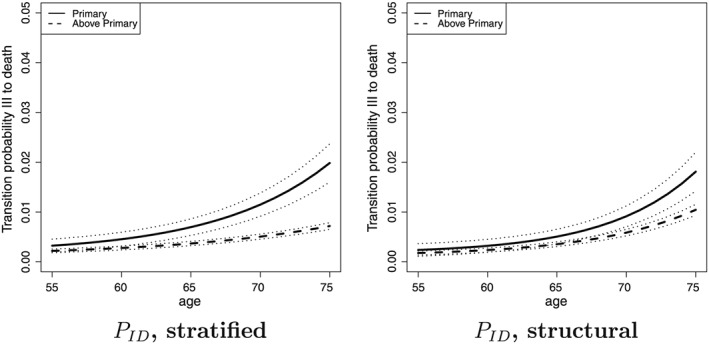
Transition probability over a 1‐year period (and the 95% confidence intervals) from hospitalized to death by education level (stratified and structural model)

Similar to the computation for the basic model, we use the estimated transition intensities from Table 3 to compute the transition probabilities. The left panel of Figure 2 shows that the transition probability from hospital to death by education becomes less distinct and is insignificant for young (below 70 years old) people when we condition on individual characteristics and hospital diagnosis (see Table C.1 in the online appendix). Still, individuals with only primary education have a higher probability to die within a year of hospital admittance when they are older than 70 years. Hence, we cannot reject Hypothesis 1 and confirm earlier findings that education is associated with improved efficiency of health investment, at least for those over 70 years old.

### 5.3 Structural model including intelligence

Next, we estimate the full structural model in which both the education choice and the transition rates depend on latent intelligence, corresponding to Eq. (5) in the theoretical framework. Table 4 reports the parameter estimates of the structural model. The coefficients for the control variables are very similar to the estimated coefficients for the transition rates in the stratified model in Table 3 and are therefore only reported in online Appendix Table C.2. Intelligence significantly affects the educational choice, and virtually all transition rates. Intelligence does not significantly affect the transition *rate* from hospitalized to death but recall that the transition *probability* depends also on the transition rates from the healthy state (Eq. (3)), for which the coefficient of intelligence is always in the expected direction and with one exception statistically significant.

**Table 4:** Parameter estimates **structural** model by education level

	Primary education		Above primary	
Transition rates *from hospitalized* ^b^				
	to healthy	to death	to healthy	to death
Intelligence	0.092^+^	0.238	0.116^∗∗^	0.129
	(0.044)	(0.183)	(0.038)	(0.138)
(log) constant	−1.576	−7.983	−1.837	−8.626
	(0.228)	(1.157)	(0.074)	(0.715)
Transition rates *from healthy*				
	to hospitalized	to death	to hospitalized	to death
Intelligence	−0.537^∗∗^	−0.142	−0.561^∗∗^	−0.649^∗∗^
	(0.159)	(0.137)	(0.161)	(0.196)
(log) constant	−3.779	−15.990	−3.396	−14.901
	(0.366)	(2.164)	(0.088)	(1.069)
Age	−	0.159	−	0.137
		(0.031)		(0.016)
Educational choice				
	Education^c^	Raven test^d^		
Intelligence	0.137^+^	1		
	(0.063)			
Measurement system				
*θ*_1_			−5.310
			(1.525)
*θ*_2_			0.426
			(0.129)
*θ*_3_			−2.628
			(0.758)
*p*_1_			0.012
			(0.003)
*p*_2_			0.871
			(0.002)
*p*_3_			0.118
			(0.015)

^b^Duration time from hospitalized is days since hospital admission.

^c^Education choice probit model.

^d^IQ‐measurement linear model, centered around IQ = 100.^+^ *p* < 0.05 and^∗∗^ *p* < 0.01.

The transition probabilities from hospital to death (and the 95% confidence intervals) are depicted in the right panel of Figure 2. We see that the difference between the two education groups has dropped after accounting for the effect of intelligence. The difference between the education levels is now for the whole age range insignificant (see Table C.1 in the online Appendix). Because the only difference between the left and right panel of Figure 2 is the addition of intelligence in the right panel, we conclude that accounting for intelligence removes most of the difference in the efficiency of health investment between higher and lower educated individuals.

### 5.4 Heterogeneity in diagnosis

**Figure 3:**
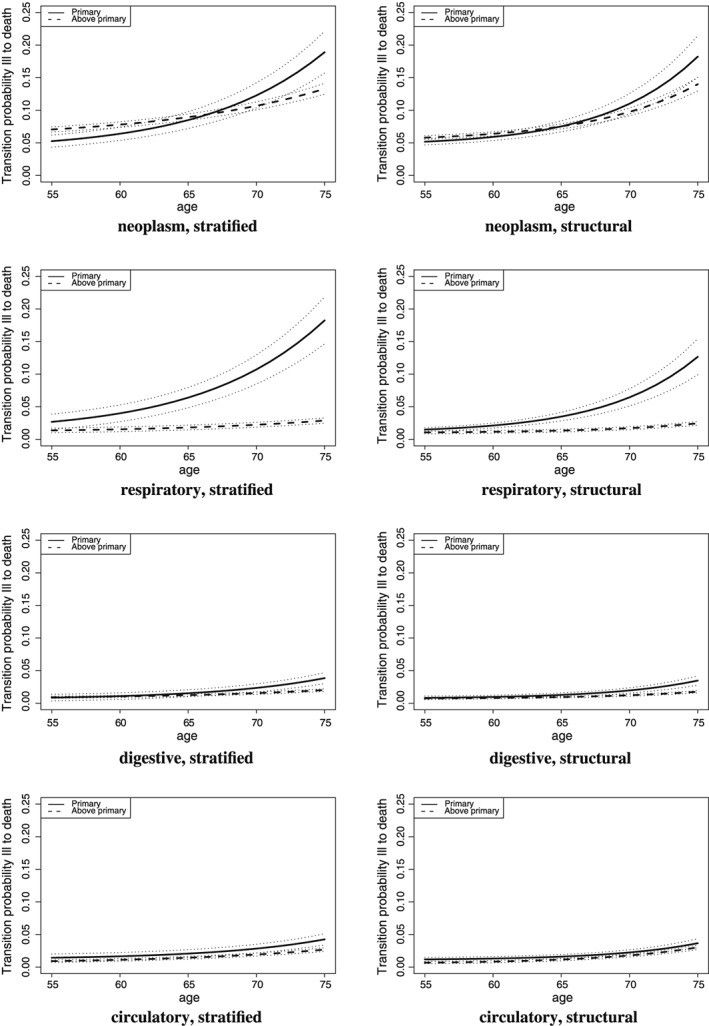
Transition probability from hospitalized to death over a 1‐year period by age and education level (stratified and structural model): DIAGNOSES

We included four different diagnoses at hospital admission in our model. Figure 3 shows the transition probability to die within 1 year after admission for these four different diagnoses. When people enter hospital and are diagnosed with cancer, survival is the same for higher and lower educated individuals. The small efficiency gain of the higher educated individuals at higher ages is removed after controlling for intelligence. On the contrary, for respiratory diseases (COPD and pneumonia), we find a large educational gain of survival after hospitalization, especially at later ages, which is only marginally reduced after controlling for intelligence. A 75‐year‐old individual with only primary education admitted to hospital with a respiratory diseases has a 13% chance to die within a year, while a higher educated individual aged 75 years with a respiratory disease has only 3% chance to die. Digestive and circulatory diseases have much lower mortality and show only a marginal gain in health efficiency by education.

This evidence suggests that when confronted with a respiratory disease, the higher educated individuals are in fact more efficient users of health investment. However, this conclusion is contingent on the assumption that there is no variation in the type and severity of illness within the broader group of respiratory admissions. Unfortunately, the sample size does not permit controlling for even finer levels of diagnoses. Nonetheless, we did check the exact International Classification of Diseases 9 codes within the larger diagnose groups; see Table 5. It follows that for circulatory and digestive diseases, the sub‐diagnoses are very similar across educational groups (e.g., coronary atherosclerosis or angina pectoris in the case of circulatory diseases and inguinal hernia and gallbladder in case of digestive diseases). The differences in proportions across educational groups are all insignificant at 5%, except for one specific code of digestive disease.

**Table 5:** ICD 9 Codes for broader class of diagnoses

ICD 9 code	Diagnosis	Primary education	Above primary
**Neoplasms**		*N* = 122	*N* = 699
154	Rectum cancer		15%^∗^
162	Lung cancer	10%	6%
174	Breast cancer (female)		7%
197	Metastasis to respiratory and digestive systems	8%	14%^∗^
198	Metastasis to kidney	38%	3%^∗^
**Circulatory diseases**		*N* = 191	*N* = 860
410	Acute myocardial infaction	6%	11%
411	Postmyorcardial infarction syndrome	10%	7%
413	Angina pectoris	10%	10%
414	Atherosclerosis	19%	22%
427	Atrial fibrillation	10%	9%
428	Heart failure	6%	3%
454	Varicose veins		5%
**Respiratory diseases**		*N* = 55	*N* = 138
473	Chronic sinusitis		7%
478	Other diseases of upper respiratory tract		14%^∗^
486	Pneumonia		12%
491	Chronic bronchitis	16%	^∗^
492	Emphysema	16%	^∗^
496	COPD (unspecified)	20%	14%
**Diseases of the digestive system**		*N* = 70	*N* = 354
550	Inguinal hernia	13%	22%
553	Other hernia of abdominal cavity	16%	8%^∗^
562	Diverticula of intestine		5%
574	Gallbladder		16%

Note: Specific ICD 9 codes for the larger group of diagnoses. Note that because of privacy regulations, we are not allowed to reveal the percentages for which the cell count is less than 10. We did take these percentages into account when testing for significant differences. A star (*) indicates that the proportions differ significantly at 5% across the two groups.ICD, International Classification of Diseases.

For neoplasms and respiratory diseases, however, differences do exist. Neoplasms among the lower‐educated individials are more often metastases. For respiratory diseases, the lower‐educated individuals are significantly more likely to be admitted for COPD, while higher educated individuals suffer more often from pneumonia and milder lung diseases. This suggests that the ‘efficiency gain’ we observed among the higher educated individuals for respiratory diseases is likely to stem largely from different diagnoses and the severity of illness rather than a true survival gain for a given diagnosis.

### 5.5 Robustness checks

In this section, we present a couple of robustness checks, results of which are all available in online Appendix C. First, there could be gender differences. The educational gains for females are indeed higher for women than for men. Still, after accounting for intelligence, these gains are insignificant.

Second, while the inclusion of the variables in *X* ^*H*^ and *X* ^*I*^, such as self‐reported health and (previous) hospital admissions, allows investigating efficiency gains for a given health status and hospital diagnosis, one may be worried that the endogeneity of these variables leads to a bias in the comparison of transition probabilities across educational groups. Therefore, we re‐estimated all models excluding these potentially endogenous variables, and results are very similar.

Third, we tested robustness to the definition of the educational choice. We re‐defined education as comprising three levels, where we split the higher educated individuals further into lower vocational education or general secondary education, and higher vocational/university. While there are some differences across the secondary and higher education groups, we find that the largest disparity is between those attending only primary education and the rest. This gives comfort that our binary representation of education is justified.

Fourth, in the base Gompertz model, we assume that the transitions are constant with respect to age, conditional on the health status and previous diagnoses of the individual. When estimating piecewise constant models without the previous diagnoses, the age dependence is positive and statistically significant. When adding the previous diagnoses, the age dependence of the transitions hazards is very limited and, in some cases, even negative. This suggests that previous diagnoses account for the age dependence in the transitions, and constant durations are a reasonable assumption. Importantly, including or excluding the duration dependence does not change any of our conclusions.

Finally, apart from the Raven test, we have estimated models in which we added an additional measurement for intelligence, namely, the vocabulary test. Because efficiency of health investment may in part derive from verbal and communication skills, it is worth extending the definition of intelligence to include this component too. The results prove robust to adding the vocabulary test.

## 6 Discussion

Higher educated individuals are healthier and live longer than their lower‐educated peers. In this paper, we formulate two testable hypotheses regarding the sources of these disparities: (i) education is associated with a higher efficiency of health investment, and (ii) conditional on intelligence, education does not improve the efficiency of health investment. In line with Kenkel (1995) and Gilleskie and Harrison (1998), we find evidence for an association between education and the efficiency of health investment: Higher educated individuals are less likely to die during middle‐age after a hospitalization. These results hold even for a given health status and given a certain diagnosis. Hence, we cannot reject our first hypothesis. Yet in contrast to these studies, we challenge the interpretation that education itself drives the efficiency of health investment: When accounting for the role of intelligence using a structural equation model, the association between education and the efficiency of health investment disappears. This suggests that intelligence accounts for a substantial proportion of the survival advantage of higher educated individuals, consistent with evidence by Conti *et al.* (2010) and Bijwaard *et al.*(2015).

Analyses investigating heterogeneity in the effects further suggest that the relative impact of education compared to intelligence is stronger for respiratory diseases. While it is tempting to attribute this to education having efficiency gains in terms of adhering to complex treatment regimens for diseases like COPD, a detailed analysis of the exact International Classification of Diseases 9 diagnoses reveals that the type of respiratory diseases contracted by higher and lower educated individuals differs. Hence, the survival gain for the higher educated individuals derives at least partially from a different severity of illness. For other diseases, the diagnoses do largely reflect the same type of diseases across educational groups, suggesting that we can interpret any survival differences as being due to health investment efficiency. For those diagnoses we find that survival gains among the higher educated individuals evaporate when conditioning on intelligence. Taken together, we conclude that conditional on intelligence, and the hospital diagnosis, education is not associated with a higher efficiency of health investment.

Most important limitation of our paper is the lack of measures for childhood health status and non‐cognitive skills. A recent survey by Heckman (2012) suggests the importance of personality traits and children's health endowment on their health outcomes as adults. The omission of these variables could therefore bias our estimates. While we do not have measures for childhood health status, it seems reasonable to assume that, conditional on cognitive abilities, the teacher's advice regarding the secondary education of the child is a proxy for non‐cognitive abilities. The results from a model allowing teacher's advice additionally to influence the transitions rates are however very close to our original results, suggesting that the omission of non‐cognitive abilities is unlikely to change our main conclusion that intelligence drives most of the efficiency gains associated with education.

In terms of policy implications, because intelligence is more important than education for the efficiency of health investment, nudging policies that alters people's behavior without forcing them (Thaler and Sunstein, 2008) may provide health improvements for all education and intelligence levels. Because nudging can change behavior non‐deliberately, thus without using the cognitive system, it could offer new possibilities for encouraging efficient use of health investment to improve survival chances among the least cognitively able. A specific example could be changing the default option for breathing exercise for COPD patients from 1 year to half a year, and excluding it from the deductible. In this way, the least cognitively able patients are stimulated and actively assisted to adhere to complex self‐care regimes without forcing them or erecting financial barriers.

## Acknowledgements

Van Kippersluis gratefully acknowledges funding from the National Institute on Aging (NIA) under grant R01AG037398 and from the Netherlands Organization of Scientific Research (NWO Veni grant 016.145.082). The authors acknowledge access to linked data resources (DO 1995‐2011, LMR 1995‐2005) by Statistics Netherlands (CBS). Further, they are grateful to two anonymous referees, Raphael Guber, attendants at the European Workshop on Econometrics and Health Economics in Paris 2015 at the NIDI/RUG workshop Early life circumstances and late life outcomes 2015, at the Multistate Event History Analysis conference in Hangzhou, the Health, Education and Retirement over the Prolonged Life Cycle conference in Vienna, the Dutch Demography day, and at a seminar at the University of Gent for helpful comments.

## Supporting information

Supporting info item

Supporting info item
