# Supplementary material for: Efficiency of Health Investment: Education or Intelligence?
Source: Health Econ. 2016 May 3;25(9):1056–72. doi: 10.1002/hec.3356 (PMC5064047; doi:10.1002/hec.3356)
Supplement: Supplementary file 1 — Supporting info item [file HEC-25-1056-s001.pdf]

Efficiency of Health Investment:
Education or Intelligence?
Appendix

A Transition probabilities

From (2) and (3) we can derive the analytical solution of the transition probabilities:

$$\begin{aligned}
P_{HH}(s,t) &= \frac{1}{\theta_1-\theta_2}\Big[(\lambda_{HI}+\lambda_{HD}+\theta_1)e^{\theta_2(t-s)}-(\lambda_{HI}+\lambda_{HD}+\theta_2)e^{\theta_1(t-s)}\Big]\\
P_{II}(s,t) &= \frac{1}{\theta_1-\theta_2}\Big[(\lambda_{HI}+\lambda_{HD}+\theta_1)e^{\theta_1(t-s)}-(\lambda_{HI}+\lambda_{HD}+\theta_2)e^{\theta_2(t-s)}\Big]\\
P_{HI}(s,t) &= \frac{\lambda_{HI}}{\theta_1-\theta_2}\Big[e^{\theta_1(t-s)}-e^{\theta_2(t-s)}\Big]\\
P_{IH}(s,t) &= \frac{\lambda_{IH}}{\theta_1-\theta_2}\Big[e^{\theta_1(t-s)}-e^{\theta_2(t-s)}\Big]
\end{aligned}$$

with two non-zero eigenvalues

$$\begin{aligned}
\theta_1 &= -\frac{1}{2}(\lambda_{HI}+\lambda_{IH}+\lambda_{HD}+\lambda_{ID})+\frac{1}{2}\sqrt{(\lambda_{HI}+\lambda_{HD}-\lambda_{IH}-\lambda_{ID})^2+4\lambda_{HI}\lambda_{IH}}\\
\theta_2 &= -\frac{1}{2}(\lambda_{HI}+\lambda_{IH}+\lambda_{HD}+\lambda_{ID})-\frac{1}{2}\sqrt{(\lambda_{HI}+\lambda_{HD}-\lambda_{IH}-\lambda_{ID})^2+4\lambda_{HI}\lambda_{IH}}
\end{aligned}$$

and

$$\theta_1-\theta_2=\sqrt{(\lambda_{HI}+\lambda_{HD}-\lambda_{IH}-\lambda_{ID})^2+4\lambda_{HI}\lambda_{IH}}$$

The probability to die at age t, the transition to death, is $1-P_{HH}(s,t)-P_{HI}(s,t)$ for an individual who is healthy at s and $1-P_{IH}(s,t)-P_{II}(s,t)$ for an individual ill at s.

Kalbfleisch et al. (1983) derive the derivatives of $P(t)$ (provided that $\theta_1 \neq \theta_2 \neq 0$). For $k \in \{HI, HD, IH, ID\}$ we have

$$\frac{\partial P(s,t)}{\partial \lambda_k} = VG_kV^{-1} \tag{A.1}$$

where $G_k(t)$ is and 3 x 3 matrix with (i,j)th element is $M_{ij}^{(k)}$ times $A_{ij}(s,t)$ with

$$A(s,t) = \begin{pmatrix} (t-s)e^{\theta_1(t-s)} & \frac{e^{\theta_1(t-s)} - e^{\theta_2(t-s)}}{\theta_1 - \theta_2} & 0 \\ \frac{e^{\theta_1(t-s)} - e^{\theta_2(t-s)}}{\theta_1 - \theta_2} & (t-s)e^{\theta_2(t-s)} & 0 \\ 0 & 0 & 0 \end{pmatrix}$$

and $M_{ij}^{(k)}$ is the (i,j)th element of $V^{-1}\big(\partial M/\partial\lambda_k\big)V$. The $\partial M/\partial\lambda_k$ matrices are very simple. e.g.

$$\frac{\partial M}{\partial \lambda_{ID}} = \begin{pmatrix} 0 & 0 & 0 \\ 0 & -1 & 1 \\ 0 & 0 & 0 \end{pmatrix}$$

B Likelihood

The likelihood contribution of the first spell (either healthy to hospitalized or healthy to death) for individual i, who is only observed (left-truncated) after t_{i0}, is given by

$$\begin{aligned} L_{i1} \quad = \quad & \int \Bigg[\Phi\big(\gamma X_i^E + \alpha_E\theta\big) \cdot \lambda_{HI}^{(1)}(t_i|X^H,\theta)^{\Delta_{HIi}} \lambda_{HD}^{(1)}(t_i|X^H,\theta)^{\Delta_{HDi}} \cdot \\ & \qquad S_H^{(1)}(t_i|X,\theta)/S_H^{(1)}(t_{i0}|X,\theta) \Bigg]^{E_i} \\ & \times \Bigg[\Phi\Big(-\gamma X_i^E - \alpha_E\theta\Big) \cdot \lambda_{HI}^{(0)}(t_i|X^H,\theta)^{\Delta_{HIi}} \lambda_{HD}^{(0)}(t_i|X^H,\theta)^{\Delta_{HDi}} \cdot \\ & \qquad S_H^{(0)}(t_i|X,\theta)/S_H^{(0)}(t_{i0}|X,\theta) \Bigg]^{1-E_i} \frac{1}{\sigma_M}\phi\Big(\frac{M_i - \delta_1 X_i^M - \alpha_M\theta}{\sigma_M}\Big)\, dH(\theta|T > t_{i0}) \end{aligned}$$

with $\Delta_{HIi} = 1$ if individual i enters hospital before dying and $\Delta_{HDi} = 1$ if individual i dies before entering hospital. The 'total' survival of individual i, the probability that he survives and stays out of hospital up till age t_i is

$$S_H^{(k)}(t|X,\theta) = \exp\Big(-\int_0^t \lambda_{HI}^{(k)}(s|X^H,\theta) + \lambda_{HD}^{(k)}(s|X^H,\theta)\,ds\Big) \quad k = 0,1$$

The distribution of the latent skills conditional on survival up to t_{i0} is

$$dH(\theta|T > t_{i0}) = \frac{\Phi\big(\gamma X_i^E + \alpha_E\theta\big)S_H^{(1)}(t_{i0}|X,\theta) + \Phi\big(-\gamma X_i^E - \alpha_E\theta\big)S_H^{(0)}(t_{i0}|X,\theta)h(\theta)}{\int \Phi\big(\gamma X_i^E + \alpha_E\theta\big)S_H^{(1)}(t_{i0}|X,\theta) + \Phi\big(-\gamma X_i^E - \alpha_E\theta\big)S_H^{(0)}(t_{i0}|X,\theta)h(\theta)\,d\theta}$$

The second spell in the multistate model (only for those who have not died) is either from hospitalized back to healthy or from hospitalized to death. Let $\tau_{i1} = t_{2i} - t_{1i}$,

the time since entry to the hospital. Then, the likelihood contribution of the second spell is

$$L_{i2} = \frac{\int f_2(\tau_{i1}|t_{i1},X,\theta)\Big[\Phi\big(\gamma X_i^E+\alpha_E\theta\big)S_H^{(1)}(t_{i1}|X,\theta)+\Phi\big(-\gamma X_i^E-\alpha_E\theta\big)S_H^{(0)}(t_{i1}|X,\theta)\Big]h(\theta)\,d\theta}{\int\Big[\Phi\big(\gamma X_i^E+\alpha_E\theta\big)S_H^{(1)}(t_{i1}|X,\theta)+\Phi\big(-\gamma X_i^E-\alpha_E\theta\big)S_H^{(0)}(t_{i1}|X,\theta)\Big]h(\theta)\,d\theta}$$

with

$$\begin{aligned} f_{i2}(\tau_{i1}|t_{i1},X,\theta) = \\ &\Big[\lambda_{IH}^{(1)}(\tau_{i1}|X^I,t_{i1},\theta)^{\Delta_{IHi}}\lambda_{ID}^{(1)}(\tau_{i1}|X^I,t_{i1},\theta)^{\Delta_{IDi}}S_I^{(1)}(t_{i2}|X,\theta)/S_I^{(1)}(t_{i1}|X,\theta)\Big]^{E_i} \\ \times &\Big[\lambda_{IH}^{(0)}(\tau_{i1}|X^I,t_{i1},\theta)^{\Delta_{IHi}}\lambda_{ID}^{(0)}(\tau_{i1}|X^I,t_{i1},\theta)^{\Delta_{IDi}}S_I^{(0)}(t_{i2}|X,\theta)/S_I^{(0)}(t_{i1}|X,\theta)\Big]^{1-E_i}\end{aligned}$$

with $\Delta_{IHi}=1$ if individual i leaves hospital before dying and $\Delta_{IDi}=1$ if individual i dies in hospital and for $k=0,1$

$$S_I^{(k)}(t_{i2}|X,\theta)=S_H^{(k)}(t_{i1}|X,\theta)\exp\Big(-\int_{t_{i1}}^{t_{i1}}\lambda_{IH}^{(k)}(s|X^I,\theta)+\lambda_{ID}^{(k)}(s|X^I,\theta)\,ds\Big)$$

The (possible) third spell in the multistate model is either from healthy back to hospitalized or from healthy to death. Then, the likelihood contribution of the third spell is

$$L_{i3}=\frac{\int f_3(t_{i3}|\theta)\Big[\Phi\big(\gamma X_i^E+\alpha_E\theta\big)S^{(1)}(t_{i2}|X,\theta)+\Phi\big(-\gamma X_i^E-\alpha_E\theta\big)S^{(0)}(t_{i2}|X,\theta)\Big]h(\theta)\,d\theta}{\int\Big[\Phi\big(\gamma X_i^E+\alpha_E\theta\big)S^{(1)}(t_{i2}|X,\theta)+\Phi\big(-\gamma X_i^E-\alpha_E\theta\big)S^{(0)}(t_{i2}|X,\theta)\Big]h(\theta)\,d\theta}$$

with

$$\begin{aligned} f_3(t_{i3}|t_{i2},X,\theta) = \\ &\Big[\lambda_{HI}^{(1)}(t_{i3}|X,\theta)^{\Delta_{HIi}}\lambda_{HD}^{(1)}(t_{i3}|X,\theta)^{\Delta_{HDi}}S^{(1)}(t_{i3}|X,\theta)/S^{(1)}(t_{i2}|X,\theta)\Big]^{E_i} \\ &\times\Big[\lambda_{HI}^{(0)}(t_{i3}|X,\theta)^{\Delta_{HIi}}\lambda_{HD}^{(0)}(t_{i3}|X,\theta)^{\Delta_{HDi}}S^{(0)}(t_{i3}|X,\theta)/S^{(0)}(t_{i2}|X,\theta)\Big]^{1-E_i}\end{aligned}$$

$\Delta_{HIi}=1$ if individual i enters (for the second time) hospital before dying and $\Delta_{HDi}=1$ if individual i dies before entering hospital (for the second time) and for $k=0,1$

$$S^{(k)}(t_{i3}|X,\theta)=S_I^{(k)}(t_{i2}|X,\theta)\frac{S_{HI}^{(k)}(t_{i3}|X,\theta)}{S_{HI}^{(k)}(t_{i2}|X,\theta)}\frac{S_{HD}^{(k)}(t_{i3}|X,\theta)}{S_{HD}^{(k)}(t_{i2}|X,\theta)}$$

The likelihood contributions for fourth and later spells are similar. The full likelihood (of individual i) is the product of all these terms, L_{i1},L_{i2},L_{i3}, etc.

C Additional Tables

Table C.1: Transition probabilities within one year by education

	$P_{ID}(t)$		$P_{IH}(t)$		$P_{HD}(t)$		$P_{HH}(t)$	
	(0)[a]	(1)[b]	(0)[a]	(1)[b]	(0)[a]	(1)[b]	(0)[a]	(1)[b]
age	*model without covariates*							
55	3.9%	2.7%*	95.8%	97.1%*	0.7%	0.4%*	99.0%	99.4%*
60	4.2%	2.8%*	95.5%	97.0%*	1.0%	0.6%*	98.7%	99.2%*
65	4.7%	3.2%*	95.0%	96.6%*	1.5%	1.0%*	98.2%	98.8%*
70	5.6%	3.7%*	94.1%	96.1%*	2.5%	1.6%*	97.2%	98.2%*
75	7.3%	4.7%*	92.4%	95.1%*	4.3%	2.6%*	95.4%	97.2%*
age	*Stratified model*, reference individual							
55	0.3%	0.2%	99.6%	99.7%	0.2%	0.1%	99.8%	99.8%
60	0.5%	0.3%	99.5%	99.7%	0.3%	0.2%	99.6%	99.8%
65	0.7%	0.4%	99.2%	99.6%	0.5%	0.3%	99.4%	99.7%
70	1.1%	0.5%*	98.8%	99.4%*	1.0%	0.4%*	98.9%	99.5%*
75	2.0%	0.7%*	97.9%	99.2%*	1.9%	0.6%*	98.1%	99.3%*
age	*Structural model*, reference individual							
55	0.2%	0.2%	99.7%	99.8%	0.0%	0.0%	99.9%	99.9%
60	0.3%	0.2%	99.6%	99.7%	0.2%	0.1%	99.8%	99.8%
65	0.5%	0.4%	99.5%	99.6%	0.3%	0.2%	99.6%	99.7%
70	0.9%	0.6%	99.1%	99.4%	0.8%	0.5%	99.2%	99.5%
75	1.8%	1.0%	98.2%	98.9%	1.7%	0.9%	98.3%	99.0%

[a] Transition probability for $E = 0$, primary education.
[b] Transition probability for $E = 1$, above primary education.
$^{*}p < 0.05$ of difference.

Table C.2: Parameter estimates structural model by education level, transition rates *from hospitalized*

	Primary education		Above primary	
	to healthy	to death	to healthy	to death
Intelligence	0.092^{+}	0.238	0.116^{**}	0.129
	(0.044)	(0.183)	(0.038)	(0.138)
Male	−0.062	0.062	0.056	0.095
	(0.116)	(0.594)	(0.051)	(0.336)
Child is working - base is “No”				
Yes	-0.494^{**}	0.246	0.084	−0.669
	(0.121)	(0.657)	(0.057)	(0.459)
Missing	−0.068	−0.320	0.045	−0.999
	(0.229)	(1.179)	(0.087)	(0.669)
Birthrank - base is “First”				
Second	−0.066	–	−0.100	0.829
	(0.205)		(0.074)	(0.458)
Third or Fourth	−0.096	–	-0.176^{**}	−0.030
	(0.185)		(0.069)	(0.493)
Fifth or higher	−0.045	–	-0.187^{**}	0.214
	(0.187)		(0.067)	(0.462)
Missing	−0.288	–	-0.330^{**}	0.593
	(0.279)		(0.123)	(0.847)
Health status in 1993 - base is “good”				
Poor health	−0.153	0.079	-0.196^{**}	−0.270
	(0.135)	(0.669)	(0.066)	(0.440)
Previous hospitalization and last diagnosis				
Repeated admittance	0.127	1.562	0.073	0.736
	(0.124)	(0.845)	(0.055)	(0.389)
Neoplasm	−0.297	1.534^{+}	-0.263^{**}	2.740^{**}
	(0.188)	(0.670)	(0.071)	(0.504)
Circulatory	0.047	0.436	−0.074	0.648
	(0.139)	(0.793)	(0.061)	(0.581)
Respiratory	−0.309	–	0.178	1.572^{+}
	(0.203)		(0.120)	(0.738)
Digestive	0.030	–	0.247^{**}	1.288
	(0.171)		(0.080)	(0.676)
Acute	-0.406^{**}	1.352	-0.340^{**}	1.458^{**}
	(0.107)	(0.779)	(0.049)	(0.368)
(log) constant	−1.576	−7.983	−1.837	−8.626
	(0.228)	(1.157)	(0.074)	(0.715)

[b] Duration time from hospitalized is days since hospital admission.

$^{+}p < 0.05$ and $^{**}p < 0.01$

Table C.2: Parameter estimates structural model by education level, transition rates *from healthy*

	Primary education		Above primary	
	to hospitalized	to death	to hospitalized	to death
Intelligence	-0.537^{**}	-0.142	-0.561^{**}	-0.649^{**}
	(0.159)	(0.137)	(0.161)	(0.196)
Male	-0.256	0.750^{**}	0.238^{**}	0.717^{**}
	(0.147)	(0.301)	(0.067)	(0.137)
Child is working - base is "No"				
Yes	0.315	0.160	0.076	0.756^{**}
	(0.164)	(0.282)	(0.073)	(0.151)
Missing	0.072	-2.005^{+}	0.003	0.261
	(0.319)	(0.789)	(0.114)	(0.160)
Family Socioeconomic Status - base is "Low"				
Middle	-0.029	-0.035	-0.224^{**}	-0.012
	(0.184)	(0.339)	(0.065)	(0.142)
High	-0.029	-0.035	0.198	0.487
	(0.184)	(0.339)	(0.195)	(0.371)
Missing	-0.698^{+}	-0.462	0.010	0.535
	(0.328)	(0.661)	(0.153)	(0.334)
Birthrank - base is "First"				
Second	0.758^{+}	-0.355	0.107	-0.011
	(0.302)	(0.440)	(0.096)	(0.192)
Third or Fourth	1.034^{**}	-0.117	0.105	-0.157
	(0.286)	(0.413)	(0.085)	(0.183)
Fifth or higher	0.659^{+}	0.073	0.113	-0.343
	(0.265)	(0.383)	(0.086)	(0.189)
Missing	1.368^{**}	1.375^{+}	-0.103	-0.835^{+}
	(0.437)	(0.651)	(0.166)	(0.396)
Health status in 1993 - base is "good"				
Poor health	0.739^{**}	-0.352	0.463^{**}	0.350
	(0.221)	(0.535)	(0.089)	(0.204)
Missing	-0.158	0.590	0.077	0.160
	(0.174)	(0.318)	(0.069)	(0.150)
Hospitalization and last diagnosis				
Has been in hospital	0.397^{+}	0.364	0.849^{**}	0.278
	(0.203)	(0.369)	(0.071)	(0.171)
Neoplasm	1.151^{**}	1.871^{**}	1.184^{**}	2.073^{**}
	(0.254)	(0.553)	(0.100)	(0.188)
Circulatory	0.555^{**}	0.104	0.636^{**}	0.732^{**}
	(0.185)	(0.438)	(0.083)	(0.198)
Respiratory	0.248	1.663^{**}	0.254	0.204
	(0.272)	(0.545)	(0.181)	(0.449)
Digestive	0.394	0.168	-0.053	-0.102
	(0.245)	(0.560)	(0.124)	(0.288)
(log) constant	-3.779	-15.990	-3.396	-14.901
	(0.366)	(2.164)	(0.088)	(1.069)
Age	–	0.159	–	0.137
		(0.031)		(0.016)

[a] Duration time from healthy is years since birth.
$^{+}p < 0.05$ and $^{**}p < 0.01$

Table C.2: Parameter estimates structural model by education level (continued)

	Education[c]	Raven test[d]
Intelligence	0.137^{+}	1
	(0.063)	
Male	-0.252^{**}	-0.887
	(0.083)	(0.528)
Child is working - base is "No"		
Yes	-0.207^{+}	-3.767^{**}
	(0.091)	(0.627)
Missing	-0.281^{+}	-1.103
	(0.137)	(0.899)
Family Socioeconomic Status - base is "Low"		
Middle	0.361^{**}	2.570^{**}
	(0.094)	(0.543)
High	0.396	4.242^{**}
	(0.453)	(1.636)
Missing	-0.511^{**}	-4.342^{**}
	(0.175)	(1.294)
Birthrank - base is "First"		
Second	-0.137	0.468
	(0.122)	(0.785)
Third or Fourth	-0.074	-0.263
	(0.113)	(0.733)
Fifth or higher	-0.057	-3.053^{**}
	(0.111)	(0.729)
Missing	0.104	-0.654
	(0.304)	(1.469)
School religion - base is "Catholic"		
Protestant	0.311^{**}	0.626
	(0.106)	(0.682)
Other	0.388^{+}	5.051^{**}
	(0.195)	(1.124)
Number of teachers - base is "5-8 teachers"		
≤ 4	-0.147	-3.837^{**}
	(0.100)	(0.725)
9-12	0.058	0.410
	(0.096)	(0.631)
Missing	0.314	0.843
	(0.215)	(1.298)
Constant	2.109	3.621
	(0.206)	(0.741)

[c] Education choice probit model.

[d] IQ-measurement linear model, centered around IQ = 100.

$^{+}p < 0.05$ and $^{**}p < 0.01$

Table C.2: Parameter estimates structural model by education level (continued)

	Education[c]	Raven test[d]	θ
Teacher's advice - base is "Lower vocational school"			
Continued primary school	-0.264^{**}		
	(0.090)		
Lower general secondary school	0.459^{**}		
	(0.165)		
Higher general secondary school	0.538^{+}		
	(0.255)		
Missing	-0.543^{+}		
	(0.250)		
Repeat grade - base is "None"			
Once	-0.295^{**}		
	(0.087)		
Twice	-0.709^{**}		
	(0.118)		
Missing	0.751		
	(0.411)		
Preference of the parents - base is "Only vocational education"			
Work in own company	-0.885^{**}		
	(0.190)		
Work without education	-1.357^{**}		
	(0.185)		
Work with education	-0.921^{**}		
	(0.198)		
General secondary school	-0.345		
	(0.179)		
Missing	-0.923^{**}		
	(0.184)		
Distribution of θ			
θ_1			-5.310
			(1.525)
θ_2			0.426
			(0.129)
θ_3			-2.628
			(0.758)
p_1			0.012
			(0.003)
p_2			0.871
			(0.002)
p_3			0.118
			(0.015)

[c] Education choice probit model.
[d] IQ-measurement linear model.
$^{+}p < 0.05$ and $^{**}p < 0.01$

Table C.3: Transition probability within one year by education and diagnosis, stratified model

	$P_{ID}(t)$		$P_{IH}(t)$		$P_{HD}(t)$		$P_{HH}(t)$	
	(0)[a]	(1)[b]	(0)[a]	(1)[b]	(0)[a]	(1)[b]	(0)[a]	(1)[b]
age				diagnosis: *neoplasm*				
55	5.3%	7.1%	94.0%	91.9%	2.4%	3.2%	96.8%	95.7%
60	6.4%	7.8%	92.8%	91.2%	3.7%	4.0%	95.6%	95.0%
65	8.5%	8.9%	90.8%	90.0%	5.9%	5.2%	93.4%	93.7%
70	12.3%	10.7%	87.0%	88.3%	9.8%	7.0%	89.4%	91.9%
75	18.9%	13.3%	80.4%	85.7%	16.8%	9.8%*	82.5%	89.1%
age				diagnosis: *circulatory diseases*				
55	1.4%	0.9%	98.2%	98.6%	0.5%	0.5%	99.1%	99.1%
60	1.7%	1.1%	98.0%	98.4%	0.7%	0.7%	98.9%	98.8%
65	2.1%	1.4%	97.5%	98.1%	1.1%	1.0%	98.5%	98.5%
70	2.8%	1.9%	96.8%	97.6%	1.9%	1.5%	97.7%	98.0%
75	4.3%	2.7%	95.4%	96.9%	3.4%	2.3%	96.2%	97.2%
age				diagnosis: *respiratory diseases*				
55	2.7%	1.4%	96.2%	98.2%	2.0%	0.5%	96.9%	99.1%
60	4.0%	1.5%	94.9%	98.1%	3.3%	0.7%	95.6%	98.9%
65	6.4%	1.8%*	92.5%	97.8%*	5.8%	1.0%*	93.1%	98.6%*
70	10.7%	2.2%*	88.2%	97.4%*	10.3%	1.4%*	88.7%	98.2%*
75	18.2%	2.9%*	80.8%	96.7%*	18.0%	2.1%*	81.0%	97.5%*
age				diagnosis: *digestive diseases*				
55	0.9%	0.9%	98.9%	98.9%	0.4%	0.3%	99.3%	99.5%
60	1.1%	1.0%	98.6%	98.8%	0.6%	0.4%	99.1%	99.4%
65	1.5%	1.2%	98.2%	98.6%	1.1%	0.7%	98.6%	99.2%
70	2.3%	1.5%	97.4%	98.3%	1.9%	1.0%	97.8%	98.9%
75	3.8%	2.0%	95.9%	97.8%	3.4%	1.4%*	96.3%	98.4%

[a] Transition probability for $E = 0$, primary education.

[b] Transition probability for $E = 1$, above primary education.

Based on structural model $^{*}p < 0.05$ of difference.

Table C.4: Transition probability within one year by education and diagnosis, structural model

	$P_{ID}(t)$		$P_{IH}(t)$		$P_{HD}(t)$		$P_{HH}(t)$	
	(0)[a]	(1)[b]	(0)[a]	(1)[b]	(0)[a]	(1)[b]	(0)[a]	(1)[b]
age	diagnosis: *neoplasm*							
55	5.2%	5.8%	94.7%	93.7%	1.1%	1.7%	98.8%	97.8%
60	5.9%	6.4%	93.9%	93.1%	1.9%	2.3%	98.0%	97.2%
65	7.5%	7.5%	92.3%	92.0%	3.6%	3.5%	96.3%	96.0%
70	11.0%	9.8%	88.9%	89.8%	7.3%	5.6%	92.6%	93.6%
75	18.2%	14.0%	81.6%	85.6%	14.9%	10.4%	85.0%	89.1%
age	diagnosis: *circulatory diseases*							
55	1.1%	0.7%	98.8%	99.1%	0.2%	0.2%	99.8%	99.5%
60	1.3%	0.8%	98.6%	98.9%	0.3%	0.4%	99.6%	99.3%
65	1.6%	1.1%	98.3%	98.6%	0.6%	0.7%	99.3%	99.0%
70	2.2%	1.8%	97.7%	98.0%	1.3%	1.4%	98.7%	98.4%
75	3.7%	3.0%	96.3%	96.7%	2.8%	2.6%	97.2%	97.1%
age	diagnosis: *respiratory diseases*							
55	1.5%	1.1%	98.4%	98.8%	0.6%	0.2%	99.4%	99.7%
60	2.2%	1.2%	97.8%	98.7%	1.2%	0.3%	98.7%	99.6%
65	3.5%	1.3%*	96.5%	98.5%*	2.6%	0.5%*	97.3%	99.4%*
70	6.4%	1.7%*	93.5%	98.2%*	5.6%	0.9%*	94.3%	99.0%*
75	12.7%	2.5%*	87.3%	97.4%*	12.0%	1.6%*	87.9%	98.3%*
age	diagnosis: *digestive diseases*							
55	0.8%	0.7%	99.1%	99.2%	0.2%	0.1%	99.8%	99.8%
60	1.0%	0.8%	99.0%	99.1%	0.3%	0.2%	99.6%	99.7%
65	1.3%	0.9%	98.7%	99.0%	0.6%	0.3%	99.3%	99.6%
70	2.0%	1.2%	98.0%	98.7%	1.3%	0.6%	98.6%	99.3%
75	3.5%	1.8%*	96.5%	98.1%	2.9%	1.2%*	97.1%	98.7%

[a] Transition probability for $E = 0$, primary education.

[b] Transition probability for $E = 1$, above primary education.

Based on structural model $^{*}p < 0.05$ of difference.

Table C.5: Transition probability within one year by education and diagnosis, females

	$P_{ID}(t)$		$P_{IH}(t)$		$P_{HD}(t)$		$P_{HH}(t)$	
	(0)[a]	(1)[b]	(0)[a]	(1)[b]	(0)[a]	(1)[b]	(0)[a]	(1)[b]
age	Stratified model: reference individual							
55	0.5%	0.3%	99.4%	99.7%	0.3%	0.2%	99.6%	99.7%
60	0.7%	0.5%	99.2%	99.5%	0.6%	0.3%	99.3%	99.6%
65	1.2%	0.6%*	98.7%	99.3%	1.1%	0.5%*	98.8%	99.4%
70	2.2%	0.9%*	97.8%	99.0%*	2.1%	0.8%*	97.9%	99.1%*
75	3.8%	1.4%*	96.1%	98.6%*	3.8%	1.3%*	96.1%	98.7%*
age	Structural model: reference individual							
55	0.3%	0.3%	99.6%	99.7%	0.2%	0.1%	99.8%	99.8%
60	0.5%	0.4%	99.5%	99.6%	0.3%	0.3%	99.6%	99.7%
65	0.9%	0.6%	99.1%	99.3%	0.7%	0.5%	99.2%	99.4%
70	1.8%	1.1%	98.2%	98.8%	1.6%	1.0%	98.4%	98.9%
75	3.6%	2.1%	96.3%	97.8%	3.5%	2.0%	96.5%	97.9%
age	Structural model diagnosis: *neoplasm*							
55	6.3%	6.9%	93.6%	92.5%	1.8%	2.7%	98.1%	96.7%
60	7.9%	8.2%	92.0%	91.3%	3.3%	4.0%	96.5%	95.4%
65	11.2%	10.5%	88.7%	88.9%	6.9%	6.6%	93.0%	92.9%
70	18.1%	15.0%	81.8%	84.4%	14.3%	11.4%	85.6%	88.1%
75	31.5%	23.3%	68.4%	76.2%	28.5%	20.2%	71.4%	79.4%
age	Structural model diagnosis: *circulatory diseases*							
55	1.4%	0.9%	98.5%	98.8%	0.3%	0.4%	99.7%	99.3%
60	1.7%	1.2%	98.2%	98.5%	0.6%	0.8%	99.4%	98.9%
65	2.3%	1.9%	97.6%	97.8%	1.2%	1.5%	98.7%	98.2%
70	3.7%	3.2%	96.3%	96.5%	2.6%	2.8%	97.4%	96.9%
75	6.6%	5.8%	93.4%	93.9%	5.6%	5.5%	94.4%	94.2%
age	Structural model diagnosis: *respiratory diseases*							
55	2.2%	1.2%	97.8%	98.6%	1.2%	0.3%	98.8%	99.5%
60	3.5%	1.4%*	96.5%	98.4%	2.5%	0.5%*	97.4%	99.3%
65	6.3%	1.8%*	93.6%	98.0%*	5.4%	0.9%*	94.5%	98.9%*
70	12.3%	2.6%*	87.7%	97.2%*	11.5%	1.8%*	88.4%	98.1%*
75	24.1%	4.2%*	75.8%	95.7%*	23.7%	3.3%*	76.3%	96.5%*
age	Structural model diagnosis: *digestive diseases*							
55	1.0%	0.9%	98.9%	99.0%	0.3%	0.2%	99.7%	99.7%
60	1.3%	1.0%	98.6%	98.9%	0.6%	0.4%	99.4%	99.5%
65	2.0%	1.3%	98.0%	98.6%	1.3%	0.7%	98.7%	99.2%
70	3.4%	1.9%	96.5%	98.0%	2.7%	1.3%	97.2%	98.6%
75	6.5%	3.0%*	93.4%	96.9%	5.9%	2.4%*	94.1%	97.5%*

[a] Transition probability for $E = 0$, primary education.

[b] Transition probability for $E = 1$, above primary education.

$^{*}p < 0.05$ of difference.

Table C.6: Transition probability within one year by education and diagnosis, model with no endogenous variables (diagnoses)

	$P_{ID}(t)$		$P_{IH}(t)$		$P_{HD}(t)$		$P_{HH}(t)$	
	(0)[a]	(1)[b]	(0)[a]	(1)[b]	(0)[a]	(1)[b]	(0)[a]	(1)[b]
age	Stratified model: reference individual							
55	0.7%	0.3%	98.2%	99.5%	0.4%	0.2%	99.5%	99.7%
60	1.0%	0.5%	98.9%	99.4%	0.7%	0.3%	99.2%	99.5%
65	1.6%	0.7%*	98.3%	99.2%	1.3%	0.5%*	98.6%	99.3%
70	2.6%	1.1%*	97.3%	98.8%*	2.3%	0.9%*	97.6%	98.9%*
75	4.6%	1.8%*	95.3%	98.1%*	4.4%	1.7%*	95.5%	98.2%*
age	Structural model: reference individual							
55	0.5%	1.3%*	99.5%	98.6%*	0.1%	0.1%	99.8%	99.8%
60	0.6%	1.4%*	99.3%	98.5%*	0.3%	0.2%	99.7%	99.7%
65	1.0%	1.6%*	99.0%	98.3%*	0.7%	0.4%	99.3%	99.5%
70	1.8%	2.0%	98.2%	98.0%	1.5%	0.8%*	98.5%	99.1%
75	3.5%	2.7%	96.5%	97.3%	3.2%	1.5%*	96.8%	98.4%*

[a] Transition probability for $E = 0$, primary education.

[b] Transition probability for $E = 1$, above primary education.

$^{*}p < 0.05$ of difference.

Table C.7: Transition probability within one year by education and diagnosis, model with 3 education levels (stratified model)

	(0)[a]	(1)[b]	(2)[c]	(0)[a]	(1)[b]	(2)[c]
	reference individual					
age		$P_{ID}(t)$			$P_{IH}(t)$	
55	0.3%	0.2%	0.2%	99.6%	99.7%	99.8%
60	0.5%	0.3%	0.2%	99.5%	99.7%	99.7%
65	0.7%	0.3%	0.3%	99.2%	99.6%	99.6%
70	1.1%	0.5%*	0.5%	98.8%	99.5%*	99.4%
75	2.0%	0.7%*	0.8%	97.9%	99.3%*	99.1%
age		$P_{HD}(t)$			$P_{HH}(t)$	
55	0.2%	0.1%	0.1%	99.8%	99.8%	99.8%
60	0.3%	0.2%	0.2%	99.6%	99.8%	99.8%
65	0.5%	0.2%	0.3%	99.4%	99.7%	99.7%
70	1.0%	0.4%*	0.5%	98.9%	99.6%*	99.5%
75	1.9%	0.6%*	0.8%	98.1%	99.4%*	99.2%
	diagnosis: *neoplasm*					
age		$P_{ID}(t)$			$P_{IH}(t)$	
55	5.3%	5.7%	6.3%	94.0%	93.3%	92.4%
60	6.4%	6.6%	6.8%	92.8%	92.4%	91.9%
65	8.5%	7.8%	7.6%	90.8%	91.2%	91.1%
70	12.3%	9.8%	9.0%	87.0%	89.3%	89.8%
75	18.9%	12.6%	11.1%	80.4%	86.5%	87.6%
age		$P_{HD}(t)$			$P_{HH}(t)$	
55	2.4%	2.9%	2.5%	96.8%	96.1%	96.2%
60	3.7%	3.8%	3.0%	95.6%	95.2%	95.6%
65	5.9%	5.1%	3.9%	93.4%	93.8%	94.7%
70	9.8%	7.2%	5.4%	89.4%	91.8%	93.3%
75	16.7%	10.2%	7.7%	82.5%	88.9%	91.0%
	diagnosis: *circulatory diseases*					
age		$P_{ID}(t)$			$P_{IH}(t)$	
55	1.4%	0.8%	1.7%	98.2%	98.7%	98.0%
60	1.7%	1.0%	1.8%	98.0%	98.5%	98.0%
65	2.1%	1.4%	2.0%	97.5%	98.1%	97.8%
70	2.8%	1.9%	2.2%	96.8%	97.6%	97.6%
75	4.3%	2.7%	2.6%	95.4%	96.8%	97.2%
age		$P_{HD}(t)$			$P_{HH}(t)$	
55	0.5%	0.5%	0.3%	99.1%	99.0%	99.5%
60	0.7%	0.8%	0.4%	98.9%	98.8%	99.4%
65	1.1%	1.2%	0.6%	98.5%	98.4%	99.2%
70	1.9%	1.7%	0.8%	97.7%	97.8%	99.0%
75	3.4%	2.5%	1.2%	96.2%	97.0%	98.6%

[a] Transition probability for $E = 0$, primary education.
[b] Transition probability for $E = 1$, secondary education.
[c] Transition probability for $E = 2$, higher education.
$^{*}p < 0.05$ of difference of adjacent education levels.

Table C.7: (continued)

	(0)[a]	(1)[b]	(2)[c]	(0)[a]	(1)[b]	(2)[c]
	diagnosis: *respiratory diseases*					
age		$P_{ID}(t)$			$P_{IH}(t)$	
55	2.7%	1.2%	0.4%	96.2%	98.3%	99.6%
60	4.0%	1.5%	0.5%	94.9%	98.1%	99.4%
65	6.4%	1.8%*	0.7%	92.5%	97.8%	99.2%
70	10.7%	2.3%*	1.0%	88.2%	97.3%*	98.9%
75	18.2%	3.0%*	1.6%	80.8%	96.5%*	98.4%
age		$P_{HD}(t)$			$P_{HH}(t)$	
55	2.0%	0.6%	0.2%	96.9%	99.0%	99.7%
60	3.3%	0.8%	0.3%	95.6%	98.8%	99.6%
65	5.8%	1.1%*	0.5%	93.1%	98.4%	99.4%
70	10.3%	1.6%*	0.9%	88.7%	97.9%*	99.0%
75	18.0%	2.4%*	1.4%	81.0%	97.1%*	98.5%
	diagnosis: *digestive diseases*					
age		$P_{ID}(t)$			$P_{IH}(t)$	
55	0.9%	0.8%	0.3%	98.9%	99.0%	99.6%
60	1.1%	1.0%	0.3%	98.6%	98.8%	99.6%
65	1.5%	1.2%	0.4%	98.2%	98.6%	99.5%
70	2.3%	1.5%	0.5%*	97.4%	98.3%	99.4%
75	3.8%	2.0%	0.7%*	95.9%	97.8%	99.2%*
age		$P_{HD}(t)$			$P_{HH}(t)$	
55	0.4%	0.3%	0.1%	99.3%	99.5%	99.8%
60	0.6%	0.5%	0.1%	99.1%	99.3%	99.8%
65	1.1%	0.7%	0.2%	98.6%	99.1%	99.7%
70	1.9%	1.1%	0.3%	97.8%	98.7%	99.6%
75	3.4%	1.6%	0.5%*	96.3%	98.2%	99.4%

[a] Transition probability for $E = 0$, primary education.
[b] Transition probability for $E = 1$, secondary education.
[c] Transition probability for $E = 2$, higher education.
$^{*}p < 0.05$ of difference of adjacent education levels.

Table C.8: Transition probability within one year by education and diagnosis, model with 3 education levels (structural model)

	(0)[a]	(1)[b]	(2)[c]	(0)[a]	(1)[b]	(2)[c]
	reference individual					
age		$P_{ID}(t)$			$P_{IH}(t)$	
55	0.3%	0.1%	0.4%	99.7%	99.9%	99.5%
60	0.3%	0.1%	0.5%	99.6%	99.8%	99.5%
65	0.5%	0.2%	0.6%	99.4%	99.7%	99.4%
70	1.0%	0.3%*	0.9%	99.0%	99.6%	99.1%
75	1.9%	0.6%*	1.4%	98.1%	99.3%*	98.6%
age		$P_{HD}(t)$			$P_{HH}(t)$	
55	0.1%	0.1%	0.1%	99.9%	99.9%	99.9%
60	0.2%	0.1%	0.1%	99.8%	99.8%	99.8%
65	0.4%	0.2%	0.3%	99.6%	99.8%	99.7%
70	0.8%	0.3%*	0.5%	99.2%	99.6%	99.4%
75	1.8%	0.6%*	1.0%	98.2%	99.3%*	98.9%
	diagnosis: *neoplasm*					
age		$P_{ID}(t)$			$P_{IH}(t)$	
55	5.6%	4.2%	5.8%	94.1%	95.4%	93.8%
60	6.3%	4.6%	6.7%*	93.4%	95.0%	92.9%
65	8.0%	5.4%	8.5%*	91.7%	94.2%	91.1%*
70	11.6%	6.9%*	12.1%*	88.1%	92.7%	87.6%*
75	19.1%	9.6%*	18.9%*	80.6%	90.1%	80.8%*
age		$P_{HD}(t)$			$P_{HH}(t)$	
55	1.4%	1.3%	1.7%	98.3%	98.3%	97.9%
60	2.2%	1.7%	2.7%	97.5%	97.9%	96.9%
65	4.0%	2.5%	4.6%	95.7%	97.0%	95.0%
70	7.8%	4.1%	8.4%*	91.9%	95.5%	91.2%*
75	15.8%	6.9%*	15.6%*	84.0%	92.7%*	84.0%*
	diagnosis: *circulatory diseases*					
age		$P_{ID}(t)$			$P_{IH}(t)$	
55	1.3%	0.3%	2.2%*	98.6%	99.5%	97.7%*
60	1.4%	0.4%	2.4%*	98.5%	99.3%	97.5%*
65	1.7%	0.7%	2.8%*	98.2%	99.1%	97.0%*
70	2.3%	1.1%	3.7%*	97.5%	98.6%	96.1%*
75	3.8%	2.0%	5.5%*	96.1%	97.7%	94.3%*
age		$P_{HD}(t)$			$P_{HH}(t)$	
55	0.2%	0.2%	0.4%	99.7%	99.5%	99.4%
60	0.3%	0.3%	0.7%	99.5%	99.4%	99.2%
65	0.6%	0.6%	1.1%	99.2%	99.1%	98.7%
70	1.3%	1.1%	2.0%	98.6%	98.6%	97.8%
75	2.7%	2.0%	3.9%	97.1%	97.7%	95.9%

[a] Transition probability for $E = 0$, primary education.
[b] Transition probability for $E = 1$, secondary education.
[c] Transition probability for $E = 2$, higher education.
$^{*}p < 0.05$ of difference of adjacent education levels.

Table C.8: (continued)

	(0)[a]	(1)[b]	(2)[c]	(0)[a]	(1)[b]	(2)[c]
	diagnosis: *respiratory diseases*					
age		$P_{ID}(t)$			$P_{IH}(t)$	
55	1.5%	1.1%	0.4%	98.3%	98.7%	99.5%
60	2.1%	1.1%	0.5%	97.7%	98.6%	99.4%
65	3.5%	1.4%*	0.7%	96.3%	98.4%	99.2%
70	6.4%	1.7%*	1.1%	93.4%	98.1%*	98.8%
75	12.6%	2.2%*	1.9%	87.2%	97.6%*	98.0%
age		$P_{HD}(t)$			$P_{HH}(t)$	
55	0.6%	0.2%	0.1%	99.2%	99.6%	99.8%
60	1.2%	0.3%	0.2%	98.6%	99.5%	99.7%
65	2.6%	0.5%*	0.4%	97.2%	99.3%	99.5%
70	5.6%	0.8%*	0.8%	94.2%	99.0%*	99.1%
75	12.0%	1.4%*	1.6%	87.8%	98.5%*	98.3%
	diagnosis: *digestive diseases*					
age		$P_{ID}(t)$			$P_{IH}(t)$	
55	0.8%	0.5%	0.4%	99.1%	99.4%	99.5%
60	0.9%	0.6%	0.4%	99.0%	99.3%	99.5%
65	1.2%	0.8%	0.6%	98.6%	99.1%	99.4%
70	1.9%	1.1%	0.8%*	97.9%	98.8%	99.1%
75	3.5%	1.6%	1.3%*	96.4%	98.3%	98.6%
age		$P_{HD}(t)$			$P_{HH}(t)$	
55	0.2%	0.1%	0.1%	99.7%	99.8%	99.8%
60	0.3%	0.2%	0.1%	99.6%	99.7%	99.8%
65	0.6%	0.4%	0.3%	99.3%	99.5%	99.7%
70	1.4%	0.7%	0.5%	98.5%	99.2%	99.4%
75	2.9%	1.3%*	1.0%	97.0%	98.7%	98.9%

[a] Transition probability for $E = 0$, primary education.
[b] Transition probability for $E = 1$, secondary education.
[c] Transition probability for $E = 2$, higher education.
$^{*}p < 0.05$ of difference of adjacent education levels.

Table C.9: Transition probability within one year by education and diagnosis, model with piecewise constant age dependence from hospitalized

	$P_{ID}(t)$		$P_{IH}(t)$		$P_{HD}(t)$		$P_{HH}(t)$	
	(0)[a]	(1)[b]	(0)[a]	(1)[b]	(0)[a]	(1)[b]	(0)[a]	(1)[b]
age	Stratified model: reference individual							
55	0.3%	0.2%	99.5%	99.7%	0.2%	0.1%	99.7%	99.8%
60	0.5%	0.3%	99.5%	99.7%	0.3%	0.2%	99.7%	99.8%
65	0.7%	0.4%	99.2%	99.6%	0.5%	0.3%	99.4%	99.7%
70	1.1%	0.5%*	98.8%	99.4%	1.0%	0.4%*	98.9%	99.5%
75	2.0%	0.7%*	97.9%	99.2%*	1.9%	0.6%*	98.1%	99.3%*
age	Structural model: reference individual							
55	0.2%	0.2%	99.7%	99.8%	0.1%	0.1%	99.9%	99.9%
60	0.3%	0.2%	99.6%	99.7%	0.2%	0.1%	99.8%	99.8%
65	0.5%	0.4%	99.4%	99.6%	0.4%	0.2%	99.6%	99.7%
70	1.0%	0.6%	99.0%	99.3%	0.8%	0.5%	99.1%	99.4%
75	1.9%	1.0%	98.0%	98.9%	1.8%	0.9%	98.2%	99.0%
age	Structural model diagnosis: *neoplasm*							
55	6.1%	5.9%	93.5%	93.7%	1.7%	1.7%	97.8%	97.8%
60	6.5%	6.3%	93.3%	93.3%	2.1%	2.2%	97.6%	97.4%
65	8.4%	7.7%	91.3%	91.8%	4.2%	3.6%	95.5%	95.8%
70	12.0%	9.8%	87.7%	89.7%	8.0%	5.9%	91.7%	93.6%
75	19.5%	13.8%	80.2%	85.7%	16.0%	10.0%	83.7%	89.4%
age	Structural model diagnosis: *circulatory diseases*							
55	1.3%	0.7%	98.5%	99.0%	0.2%	0.2%	99.6%	99.5%
60	1.4%	0.8%	98.5%	98.9%	0.3%	0.4%	99.6%	99.4%
65	1.7%	1.2%	98.1%	98.5%	0.7%	0.8%	99.2%	98.9%
70	2.4%	1.8%	97.5%	97.9%	1.3%	1.4%	98.5%	98.3%
75	3.8%	3.0%	96.0%	96.7%	2.8%	2.6%	97.1%	97.0%
age	Structural model diagnosis: *respiratory diseases*							
55	1.6%	1.1%	98.1%	98.8%	0.6%	0.2%	99.1%	99.7%
60	2.2%	1.2%	97.7%	98.7%	1.2%	0.3%	98.6%	99.6%
65	3.5%	1.4%*	96.2%	98.4%	2.6%	0.5%	97.1%	99.3%
70	6.5%	1.8%*	93.3%	98.1%*	5.7%	0.9%*	94.1%	98.9%*
75	12.7%	2.5%*	87.1%	97.3%*	12.1%	1.7%*	87.7%	98.1%*
age	Structural model diagnosis: *digestive diseases*							
55	0.8%	0.7%	99.0%	99.2%	0.2%	0.1%	99.7%	99.8%
60	0.9%	0.8%	99.0%	99.1%	0.3%	0.2%	99.6%	99.7%
65	1.3%	1.0%	98.6%	98.9%	0.6%	0.3%	99.2%	99.5%
70	2.0%	1.2%	97.9%	98.7%	1.4%	0.6%	98.5%	99.3%
75	3.5%	1.8%	96.4%	98.1%	2.9%	1.2%	97.0%	98.7%

[a] Transition probability for $E = 0$, primary education.

[b] Transition probability for $E = 1$, above primary education.

$^{*}p < 0.05$ of difference.

Table C.10: Transition probability within one year by education and diagnosis, model with two IQ-tests

	$P_{ID}(t)$		$P_{IH}(t)$		$P_{HD}(t)$		$P_{HH}(t)$	
	(0)[a]	(1)[b]	(0)[a]	(1)[b]	(0)[a]	(1)[b]	(0)[a]	(1)[b]
age	Structural model: reference individual							
55	0.2%	0.2%	99.7%	99.8%	0.1%	0.1%	99.9%	99.9%
60	0.3%	0.2%	99.6%	99.7%	0.2%	0.1%	99.8%	99.8%
65	0.5%	0.4%	99.5%	99.6%	0.3%	0.2%	99.6%	99.7%
70	0.9%	0.6%	99.1%	99.4%	0.8%	0.5%	99.2%	99.5%
75	1.8%	1.0%	98.2%	98.9%	1.6%	0.9%	98.3%	99.0%
age	Structural model diagnosis: *neoplasm*							
55	4.0%	5.6%*	95.9%	93.9%*	0.9%	1.6%	99.0%	97.9%
60	4.7%	6.1%	95.2%	93.4%	1.7%	2.1%	98.2%	97.4%
65	6.4%	7.0%	93.5%	92.5%	3.4%	3.0%	96.5%	96.5%
70	9.9%	8.7%	90.0%	90.9%	7.1%	4.8%	92.8%	94.7%
75	17.1%	11.9%	82.8%	87.6%	14.6%	8.2%*	85.3%	91.3%
age	Structural model diagnosis: *circulatory diseases*							
55	1.0%	0.6%	99.0%	99.1%	0.2%	0.2%	99.8%	99.6%
60	1.1%	0.8%	98.8%	99.0%	0.3%	0.3%	99.7%	99.4%
65	1.4%	1.0%	98.5%	98.8%	0.6%	0.6%	99.4%	99.2%
70	2.1%	1.5%	97.9%	98.3%	1.3%	1.0%	98.7%	98.7%
75	3.5%	2.4%	96.5%	97.4%	2.7%	2.0%	97.3%	97.8%
age	Structural model diagnosis: *respiratory diseases*							
55	1.0%	1.0%	98.9%	98.8%	0.6%	0.2%	99.4%	99.7%
60	1.7%	1.1%	98.3%	98.8%	1.3%	0.2%*	98.7%	99.6%
65	3.2%	1.2%*	96.8%	98.6%	2.8%	0.4%*	97.2%	99.5%*
70	6.3%	1.5%*	93.7%	98.4%*	5.9%	0.7%*	94.0%	99.2%*
75	12.8%	2.1%*	87.2%	97.8%*	12.6%	1.2%*	87.4%	98.6%*
age	Structural model diagnosis: *digestive diseases*							
55	0.8%	0.7%	99.2%	99.2%	0.1%	0.1%	99.8%	99.8%
60	0.9%	0.8%	99.0%	99.1%	0.3%	0.2%	99.7%	99.7%
65	1.3%	0.9%	98.7%	99.0%	0.6%	0.3%	99.3%	99.6%
70	1.9%	1.1%	98.0%	98.8%	1.3%	0.5%	98.7%	99.4%
75	3.4%	1.5%*	96.5%	98.4%	2.8%	0.9%*	97.1%	99.0%*

[a] Transition probability for $E = 0$, primary education.
[b] Transition probability for $E = 1$, above primary education.
$^{*}p < 0.05$ of difference.

Table C.11: Transition probability within one year by education and diagnosis, model with teacher advice

	$P_{ID}(t)$		$P_{IH}(t)$		$P_{HD}(t)$		$P_{HH}(t)$	
	(0)[a]	(1)[b]	(0)[a]	(1)[b]	(0)[a]	(1)[b]	(0)[a]	(1)[b]
age	Stratified model: reference individual							
55	0.3%	0.2%	99.6%	99.7%	0.2%	0.1%	99.8%	99.8%
60	0.4%	0.3%	99.5%	99.7%	0.3%	0.2%	99.6%	99.8%
65	0.7%	0.3%	99.3%	99.6%	0.5%	0.3%	99.4%	99.7%
70	1.1%	0.5%*	98.8%	99.5%	1.0%	0.4%*	99.0%	99.5%*
75	1.9%	0.7%*	98.0%	99.3%*	1.8%	0.6%*	98.1%	99.3%*
age	Structural model: reference individual							
55	0.2%	0.2%	99.7%	99.8%	0.0%	0.0%	99.9%	99.9%
60	0.3%	0.2%	99.6%	99.7%	0.2%	0.1%	99.8%	99.8%
65	0.5%	0.3%	99.5%	99.6%	0.3%	0.2%	99.6%	99.7%
70	0.9%	0.6%	99.1%	99.4%	0.7%	0.5%	99.2%	99.5%
75	1.8%	1.0%	98.2%	98.9%	1.6%	0.9%	98.3%	99.0%
age	Structural model diagnosis: *neoplasm*							
55	4.9%	5.1%	94.9%	94.5%	1.1%	1.5%	98.5%	98.0%
60	5.6%	5.6%	94.2%	94.0%	1.9%	2.1%	97.8%	97.5%
65	7.0%	6.6%	92.7%	92.9%	3.5%	3.2%	96.3%	96.4%
70	10.3%	8.7%	89.5%	90.9%	6.9%	5.3%	92.8%	94.2%
75	17.2%	12.7%	82.6%	86.9%	14.2%	9.5%	85.6%	90.0%
age	Structural model diagnosis: *circulatory diseases*							
55	1.2%	0.6%	98.7%	99.1%	0.2%	0.2%	99.7%	99.5%
60	1.3%	0.8%	98.6%	98.9%	0.3%	0.4%	99.6%	99.3%
65	1.6%	1.1%	98.3%	98.6%	0.6%	0.7%	99.3%	99.0%
70	2.2%	1.7%	97.7%	98.0%	1.2%	1.3%	98.7%	98.4%
75	3.5%	3.0%	96.4%	96.8%	2.5%	2.6%	97.4%	97.1%
age	Structural model diagnosis: *respiratory diseases*							
55	1.4%	1.0%	98.5%	98.8%	0.6%	0.2%	99.3%	99.6%
60	2.0%	1.1%	97.9%	98.7%	1.2%	0.4%	98.7%	99.5%
65	3.3%	1.4%*	96.6%	98.5%	2.6%	0.6%*	97.3%	99.2%
70	6.2%	1.8%*	93.7%	98.0%*	5.5%	1.1%*	94.3%	98.7%*
75	12.4%	2.8%*	87.5%	97.0%*	11.8%	2.0%*	88.0%	97.8%*
age	Structural model diagnosis: *digestive diseases*							
55	0.7%	0.6%	99.2%	99.3%	0.1%	0.1%	99.8%	99.8%
60	0.8%	0.7%	99.1%	99.2%	0.3%	0.2%	99.6%	99.7%
65	1.1%	0.8%	98.8%	99.1%	0.5%	0.3%	99.4%	99.6%
70	1.7%	1.1%	98.2%	98.8%	1.1%	0.6%	98.8%	99.3%
75	3.0%	1.6%	96.9%	98.3%	2.5%	1.1%	97.4%	98.8%

[a] Transition probability for $E = 0$, primary education.

[b] Transition probability for $E = 1$, above primary education.

$^{*}p < 0.05$ of difference.
